# Supplementary material for: Cancer cell type-specific derepression of transposable elements by inhibition of chromatin modifier enzymes
Source: Commun Biol. 2025 Jul 3;8:992. doi: 10.1038/s42003-025-08413-0 (PMC12229592; doi:10.1038/s42003-025-08413-0)
Supplement: Supplementary file 2 — Supplementary Information [file 42003_2025_8413_MOESM2_ESM.pdf]

Supplementary Figures 1-21:

Cancer cell type-specific derepression of transposable elements by inhibition of chromatin modifier enzymes

Divyesh Patel[1,2,5], Ville Tiusanen[1], Konsta Karttunen[1,2], Päivi Pihlajamaa[1,3] and Biswajyoti Sahu[1,2,3,4,5#]

[1]Applied Tumor Genomics Program, Research Programs Unit, Faculty of Medicine, University of Helsinki, Helsinki, Finland

[2]iCAN Digital Precision Cancer Medicine Flagship, University of Helsinki, Helsinki, Finland

[3]Medicum, Faculty of Medicine, University of Helsinki, Helsinki, Finland

[4]Department of Medical Genetics, Institute for Cancer Research, Oslo University Hospital, Oslo, Norway

[5]Norwegian Centre for Molecular Biosciences and Medicine, Faculty of Medicine, University of Oslo, Oslo, Norway

# Corresponding author: Biswajyoti Sahu (biswajyoti.sahu@ncmbm.uio.no, biswajyoti.sahu@helsinki.fi)

Supplementary Figure 1

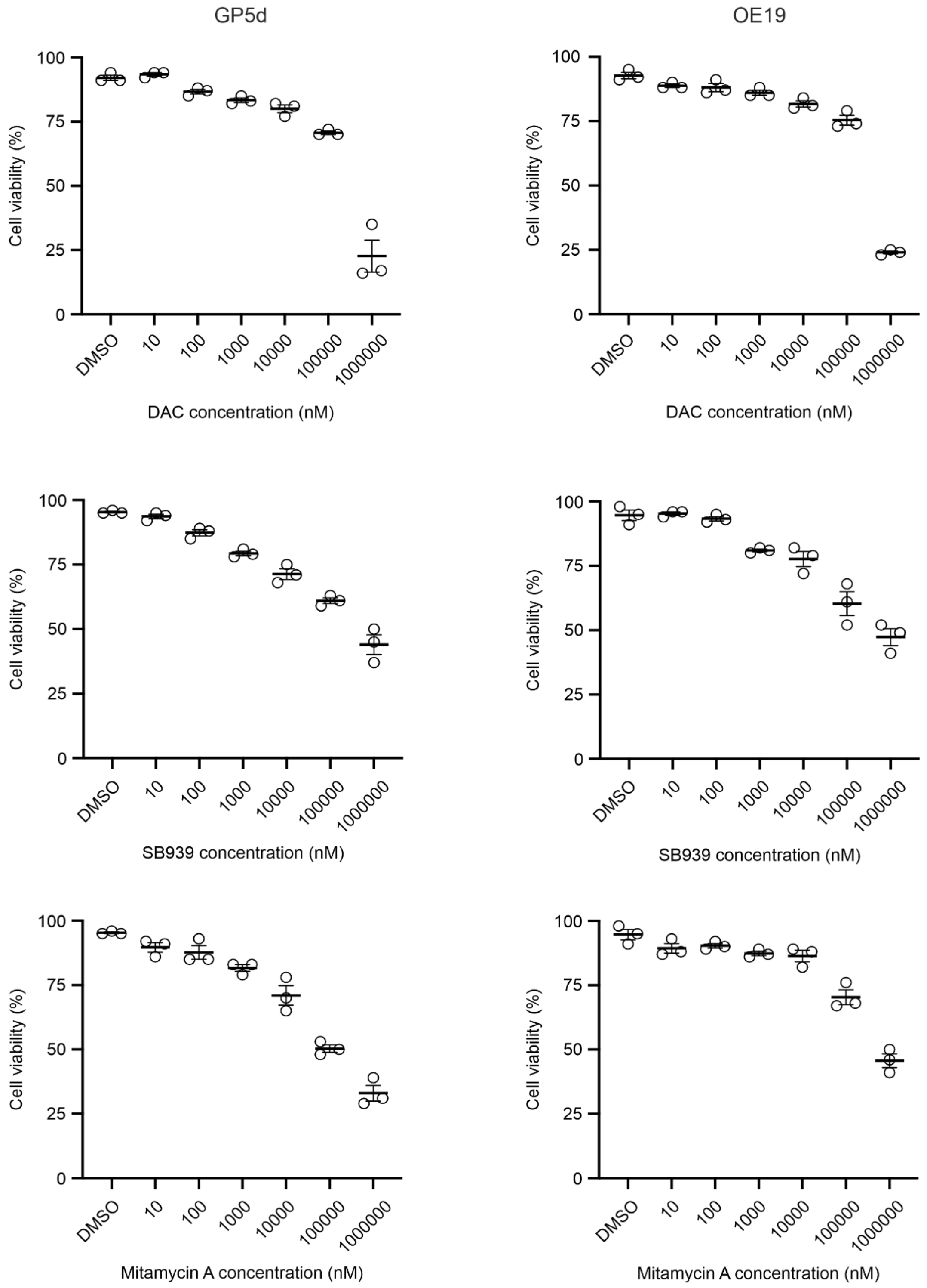

Supplementary Figure 1 | Cell viability assay for CME inhibitors. Comparison of the viability of GP5d and OE19 cells treated with DAC (DNMT inhibitor) for 72 h, SB939 (HDAC inhibitor) for 24 h and Mitramycin A (SETDB1 inhibitor) for 24 h at varying doses (10 nM-1 mM). For DNMTi treatment, media containing DAC was replenished every day. The figures show mean ± SEM values for three replicates. Of note, the treatments did not induce considerable cytotoxicity at the concentration used in this study (500 nM). Larger doses resulted in reduced cell viability comparable to previous reports, such as ref.[1-3]. The graph shows mean ± SD values for three replicates. Source data are provided as **Supplementary Data 11**.

Supplementary Figure 2

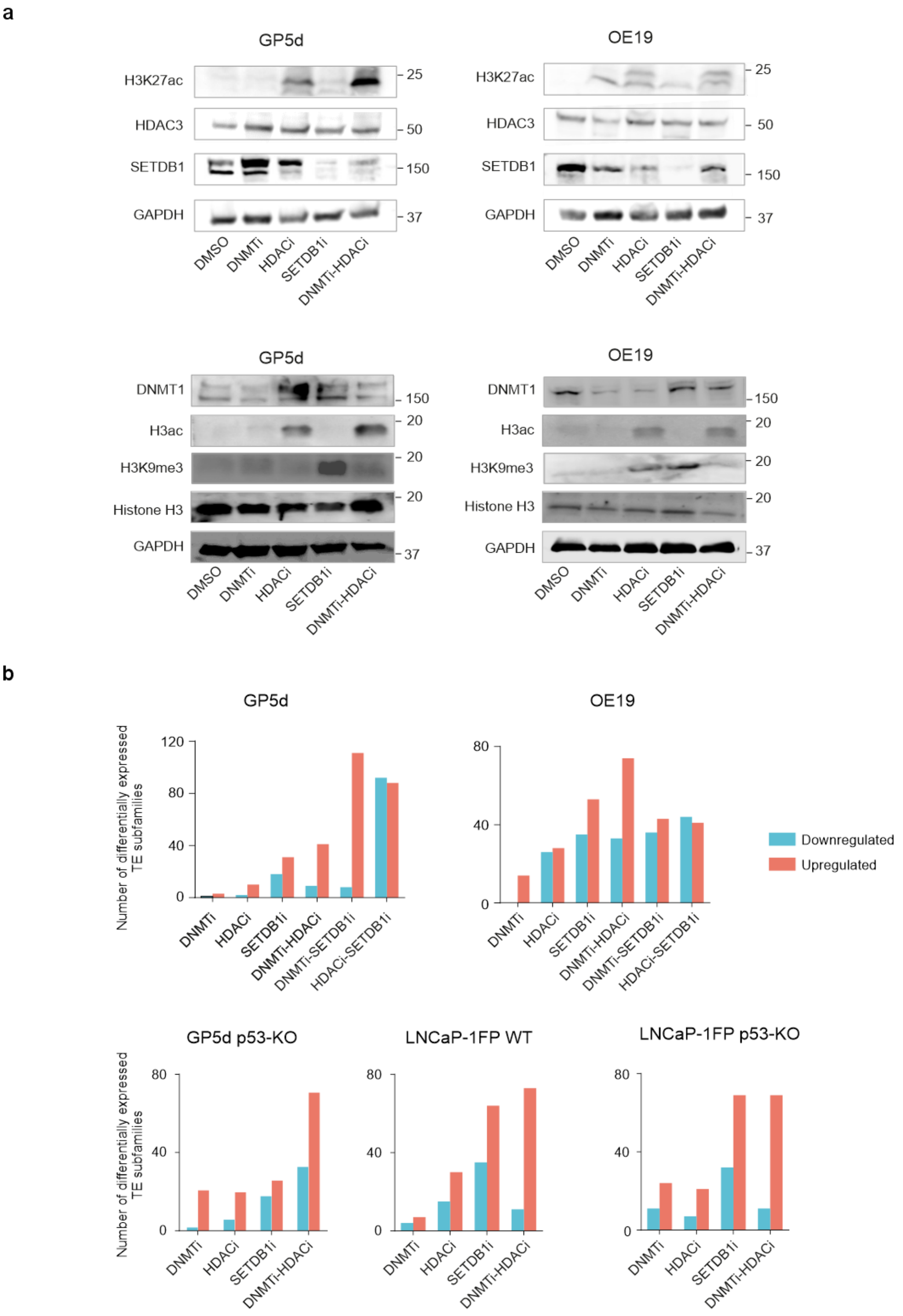

Supplementary Figure 2 | TE subfamilies are synergistically derepressed by co-inhibition of different CMEs. **a,** Western blot of H3K27ac, HDAC3, SETDB1, DNMT1, H3ac (pan-acetyl), H3K9me3, Histone H3 and GAPDH expression for GP5d and OE19 cells treated with distinct CMEs inhibitors or DMSO control. Molecular weights in kDa are marked on the right side of the blots. GAPDH and Histone H3 were used as a loading control. **b,** Number of differentially expressed TE subfamilies induced by the inhibition of CMEs in GP5d, OE19, GP5d p53-KO, LNCaP-1F5 and LNCaP-1F5 p53-KO cells. TE subfamilies meeting the threshold criteria of absolute log2 fold change (log2FC) > 1.5 and adjusted p-value < 0.05 are considered as differentially expressed. See also **Supplementary Data 1**. Source data are provided as **Supplementary Data 11**.

Supplementary Figure 3

a

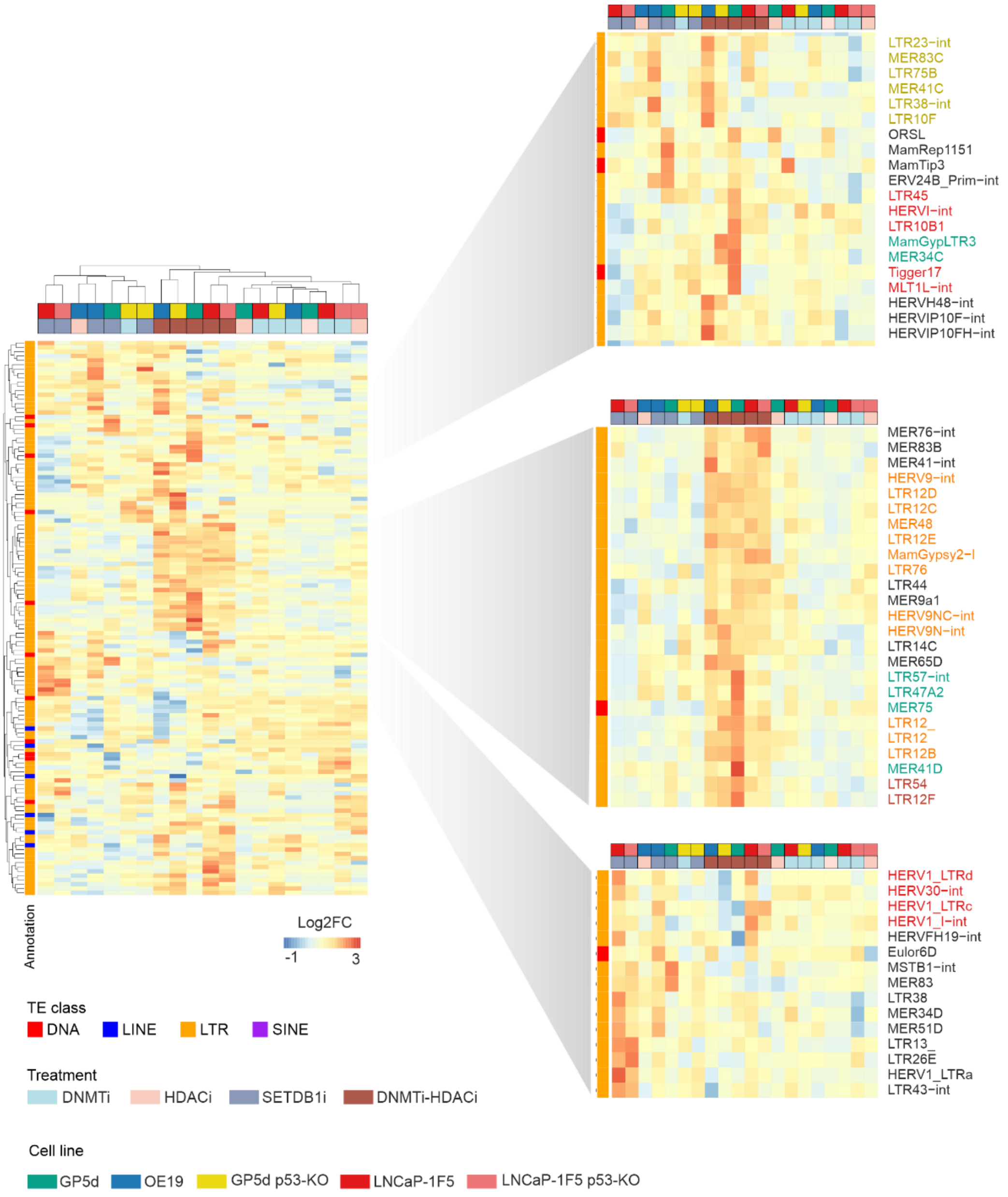

Supplementary Figure 3 | TE subfamilies are derepressed in a cell type-specific manner by inhibition of CMEs. **a,** Comparison of CME treatment-induced expression changes in TE subfamilies between five cell lines. TE subfamilies meeting the threshold criteria of absolute log2FC > 2.5 (treatment vs DMSO control) and adjusted p-value < 0.05 in at least one CMEs treatment were plotted. Rows and columns are clustered with hierarchical clustering with Euclidean distances and Ward linkage. TE subfamilies derepressed by DNMTi-HDACi treatment in all five cell lines are highlighted in the right middle panel (orange color). TE subfamilies derepressed by DNMTi-HDACi in LNCaP-1F5 WT (red color), GP5d wild-type (green color) and OE19 cells (yellow color) are highlighted in the right top, middle, and bottom panels. Source data are provided as **Supplementary Data 11**.

Supplementary Figure 4

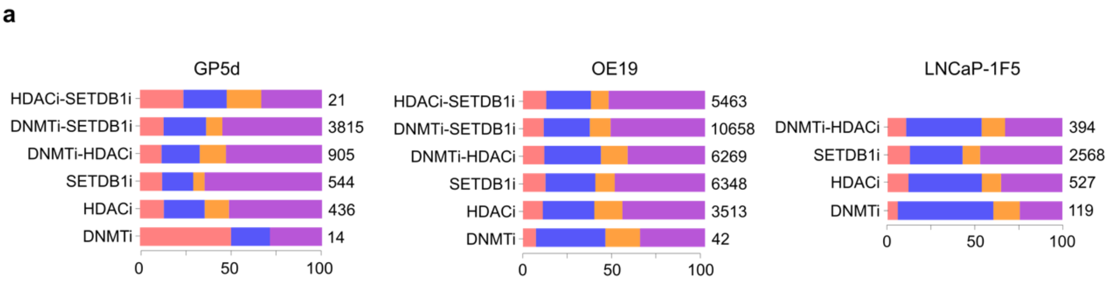

Supplementary Figure 4 | Major TE classes of TEs repressed by CME treatments. **a,** The proportion of repressed TE loci by inhibition of CMEs belonging to major TE classes in GP5d, OE19 and LNCaP-1F5 cells. Repressed TE loci were labeled by TE class and their counts presented as percentage of total. Numbers represent total repressed TE loci. Source data are provided as **Supplementary Data 11**.

Supplementary Figure 5

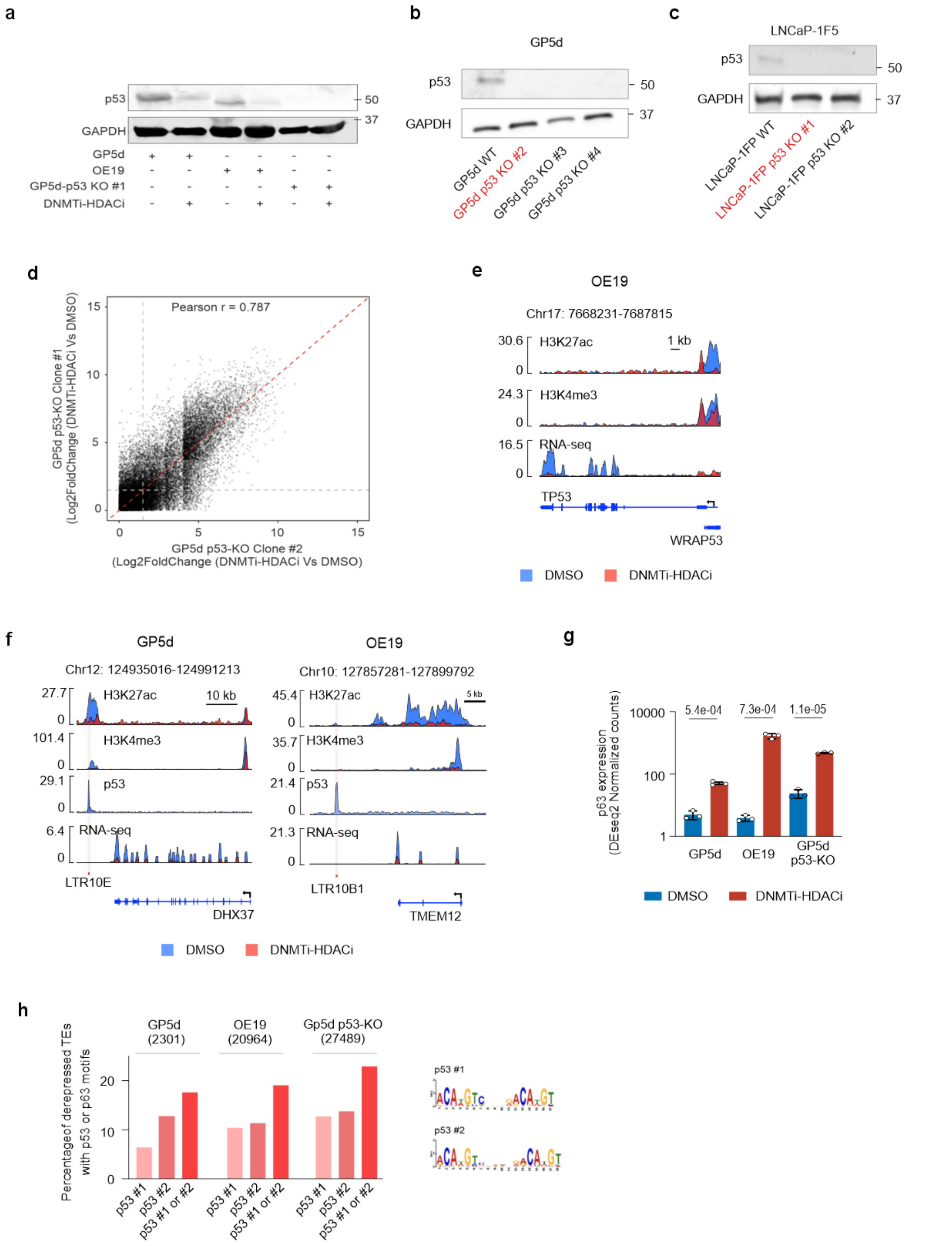

Supplementary Figure 5 | CRISPR deletion of p53 in GP5d and LNCaP-1F5 cells. a, Western blot of p53 expression for GP5d, OE19, and GP5d p53-KO #1 cells with and without DNMTi-HDACi treatments. Molecular weights in kDa are shown on the right side of the blots. We also observed a smaller p53 protein (approximately 40 kDa) in OE19 cells compared to GP5d cells (53 kDa), validating the nonsense mutation in the tetramerization domain of p53 reported earlier[4]. **b,** Western blot of p53 expression for GP5d wild-type and additional GP5d p53-KO clones (clone #2 was used for RNA-seq experiment, highlighted in red). Molecular weights are shown as in **a**. **c,** Western blot of p53 expression for LNCaP-1F5 wild-type and

LNCaP-1F5 p53-KO clones. (clone #1 was used for RNA-seq experiment, highlighted in red). Molecular weights are shown as in **a.** **d,** GP5d P53-KO clone #1 and #2 were used to compare TE derepression by DNMTi-HDACi in p53-KO GP5d cells. The scatter plot compares log2FC of TE expression (DNMTi-HDACi vs DMSO control) between the two p53-KO clones. Pearson's r is shown in the scatter plot. **e,** Genome browser snapshot of the *TP53* gene for DMSO control and DNMTi-HDACi OE19 cells. Each panel shows the ChIP-seq signals for H3K27ac and H3K4me3 and an RNA-seq signal track for both DMSO control and DNMTi-HDACi cells. **f,** Genome browser snapshot of p53-regulated *DHX37* and *TMEM12* genes in GP5d cells with and without DNMTi-HDACi. *DHX37* and*TMEM12* genes are regulated by p53-bound downstream LTR10E and LTR10B1[5]. Each panel shows the ChIP-seq signals for H3K27ac, H3K4me3, and p53 and an RNA-seq signal track for both DMSO and DNMTi-HDACi GP5d cells. Panel for p53 ChIP-seq shows signal from GP5d cells. **g,** Bar plots comparing normalized RNA-seq read counts for *p63* gene in GP5d, OE19 and p53-KO GP5d cells treated with DNMTi-HDACi or DMSO control. The graph shows mean ± SD values for three biological replicates (unpaired two-sided t-test). GP5d p53-KO clone #1 was the clone selected for further comparisons and is referred to as GP5d p53-KO further on. **h,** TEs derepressed by DNMTi-HDACi were compared for the enrichment of p53 DNA binding motifs. Bar plots compare the percentage of TEs with p53 motifs derepressed by DNMTi-HDACi. Position weight matrices for p53 motifs were extracted from ref.[6], and motif occurrence analysis was performed with FIMO (MEME suite) with default parameters. Source data are provided as **Supplementary Data 11**.

Supplementary Figure 6

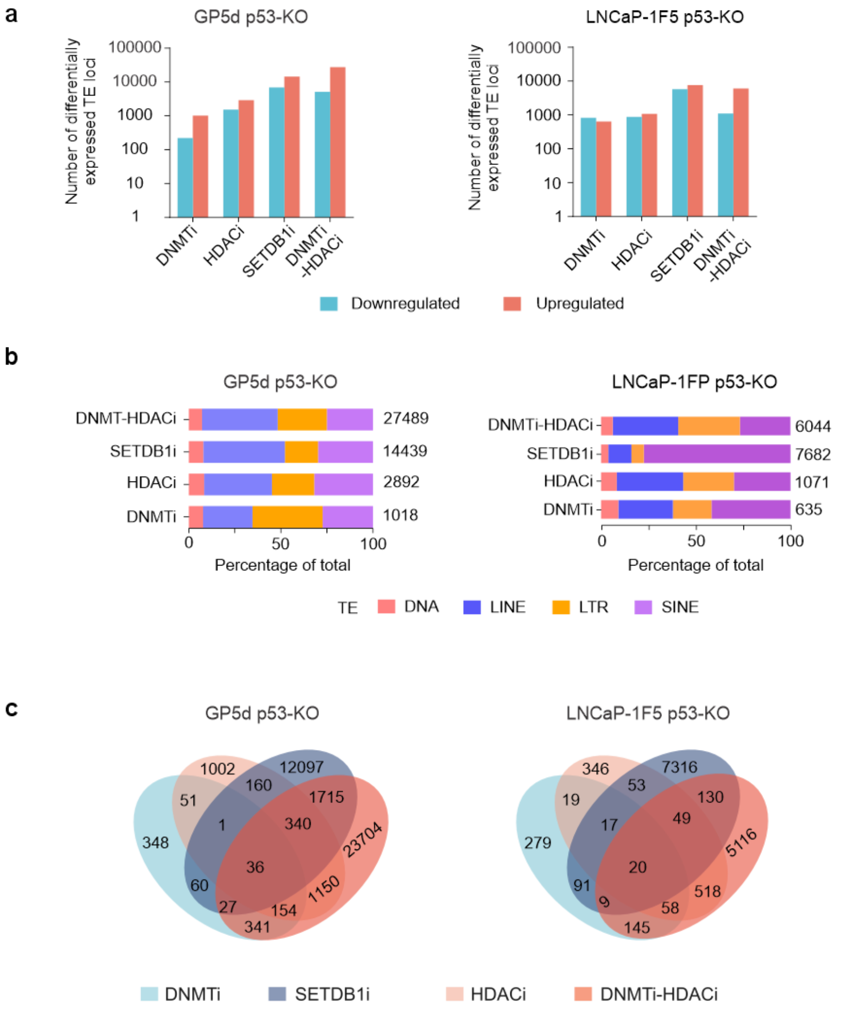

Supplementary Figure 6 | Loss of p53 results in stronger depression of TEs. a, Number of differentially expressed TE loci induced by inhibition of CMEs in GP5d p53-KO and LNCaP-1F5 p53-KO cells. TE loci meeting the threshold criteria of Log2FC > 1.5 and adjusted p-value < 0.05 compared to vehicle-treated cells are considered as differentially expressed. **b,** The proportion of derepressed TE loci by inhibition of CMEs belonging to major TE classes in GP5d p53-KO and LNCaP-1F5 p53-KO cells as a percentage of all total derepressed loci. Numbers on right represent the total count of derepressed TE loci. **c,** Overlap of derepressed individual TE loci by inhibition of CMEs in GP5d p53-KO and LNCaP-1F5 p53-KO cells. Source data are provided as **Supplementary Data 11**.

Supplementary Figure 7

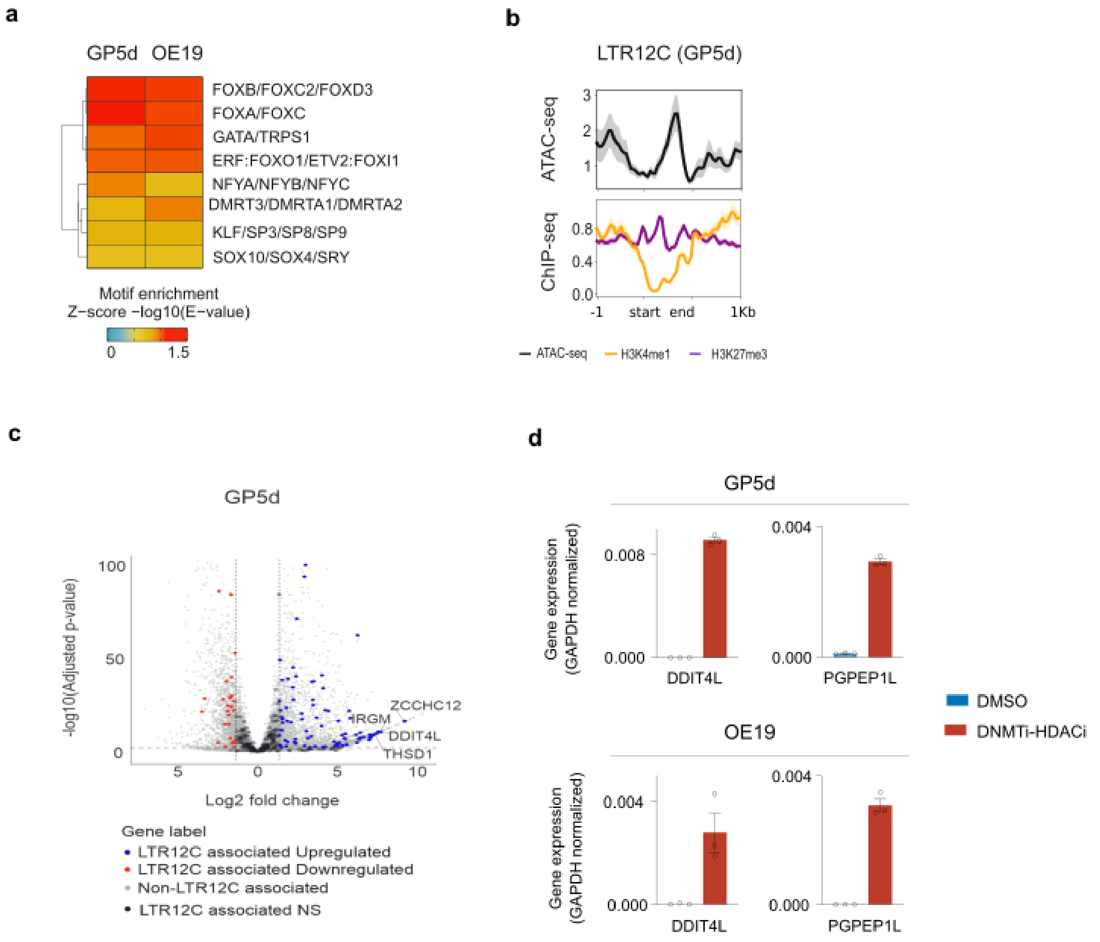

Supplementary Figure 7 | LTR12C derepressed by DNMTi-HDACi transactivates nearby genes. a, TF motif enrichment at derepressed LTR12C in DNMTi-HDACi-treated GP5d and OE19 cells. After performing motif enrichment analysis for individual motifs, similar motifs were combined into motif clusters according to ref. [7]. The representative TF families are labeled on the right. Clustering was performed using Euclidean distance and Ward's method. **b,** Metaplots of ATAC-seq and ChIP-seq for H3K27me3 and H3K4me1 in GP5d cells at LTR12C elements derepressed by co-inhibition of DNMT and HDAC (shown in **Fig. 4b**). To note, derepressed LTR12C does not show enrichment of active H3K4me3 marks as observed in OE19 cells. **c,** Volcano plot of derepressed LTR12C-associated gene expression in DNMTi-HDACi GP5d (shown in **Fig. 4b**), showing significant upregulation of genes within 50kb of the derepressed elements. Differentially expressed genes were defined by absolute Log2FC > 1.5 (DNMTi-HDACi vs DMSO cells from the same cell line) and adjusted p-value < 0.05. **d,** qRT-PCR data showing changes in mRNA expression for LTR12C-associated genes upregulated by DNMTi-HDACi treatment in GP5d and OE19 cells. Y-axis shows GAPDH normalized gene expression. *DDIT4L* and *PGPEP1L* were significantly upregulated in DNMTi-HDACi treated GP5d and OE19 cells. The graph shows mean ± SD values for three replicates. Source data are provided as **Supplementary Data 11**.

Supplementary Figure 8

a

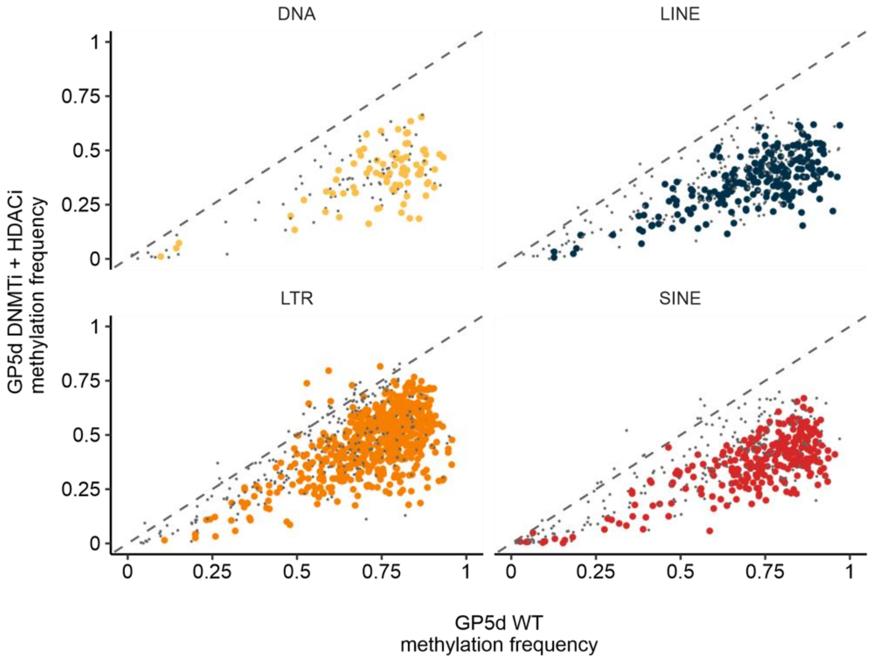

Supplementary Figure 8 | TE DNA methylation loss in DNMTi-HDACi treated GP5d cells. **a,** NaNOMe-seq was performed in untreated and DNMTi-HDACi GP5d cells. Scatter plots compare the CpG methylation frequency of all transcriptionally upregulated TEs in each TE class between the treated and untreated cells with a two-sided Fisher's test. Statistically significant loci with a BH-adjusted p-value < 0.05 are shown in color and insignificant loci are plotted with gray points. Source data are provided as **Supplementary Data 11**.

Supplementary Figure 9

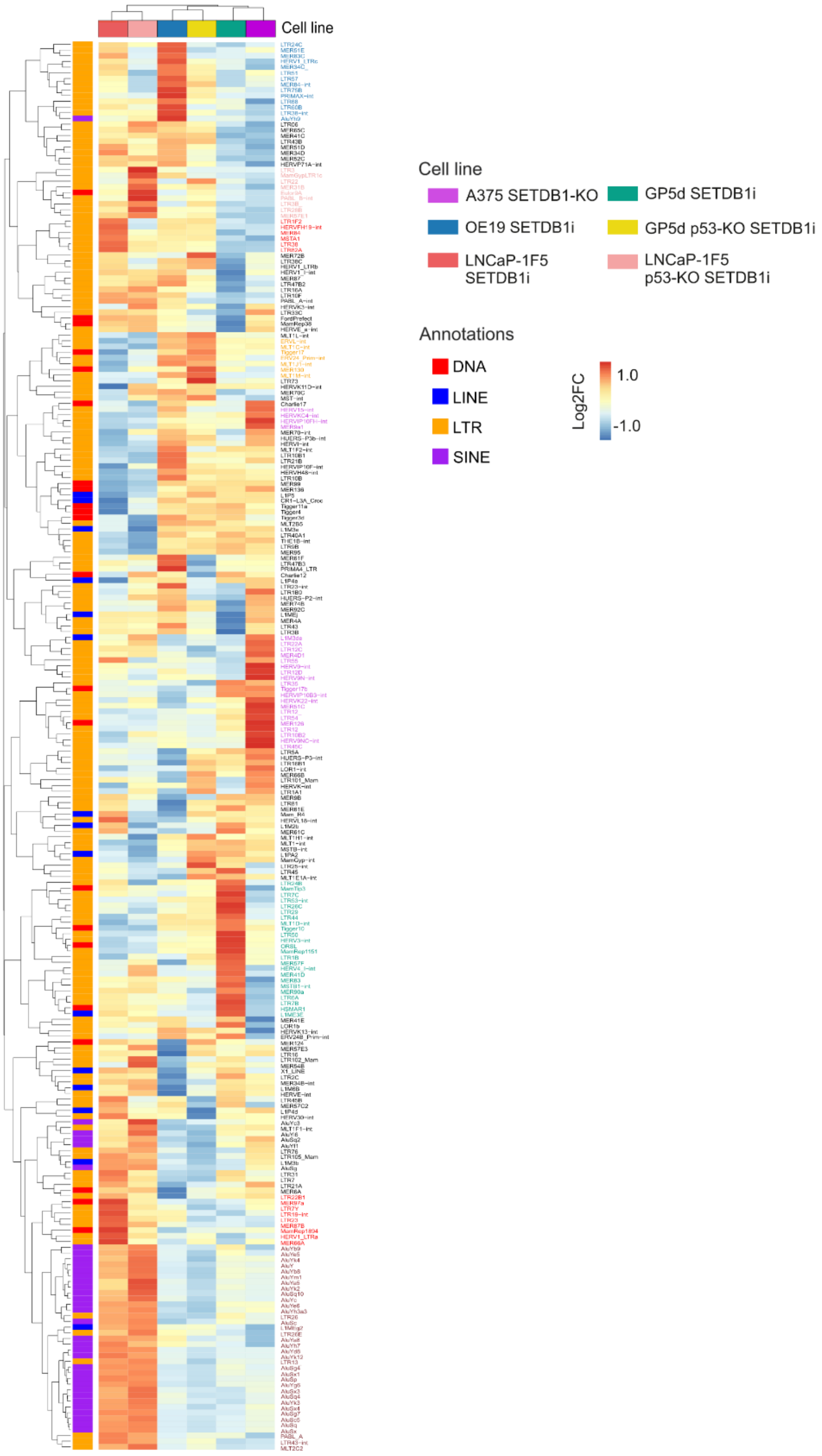

Supplementary Figure 9 | Comparison of SETDB1 KO/inhibition induced expression changes in TE subfamilies between six cell lines. TE subfamilies with absolute log2FC > 1.5 (treatment vs DMSO control or A375 SETDB1 KO vs A375) and adjusted p-value < 0.05 in at least one CME treatment were plotted. Rows and columns are clustered with hierarchical clustering. Clustering was performed using Euclidean distance and Ward's method. Cell type-specific derepressed TE subfamilies are highlighted (A375 specific in purple, GP5d specific in green, OE19 specific in blue and GP5d p53-KO specific in yellow, LNCaP-1F5 specific in red and LNCaP-1F5 p53-KO specific in pink). TE subfamilies depressed in LNCaP-1FP and LNCaP-1FP p53-KO cells are highlighted in maroon.

Supplementary Figure 10

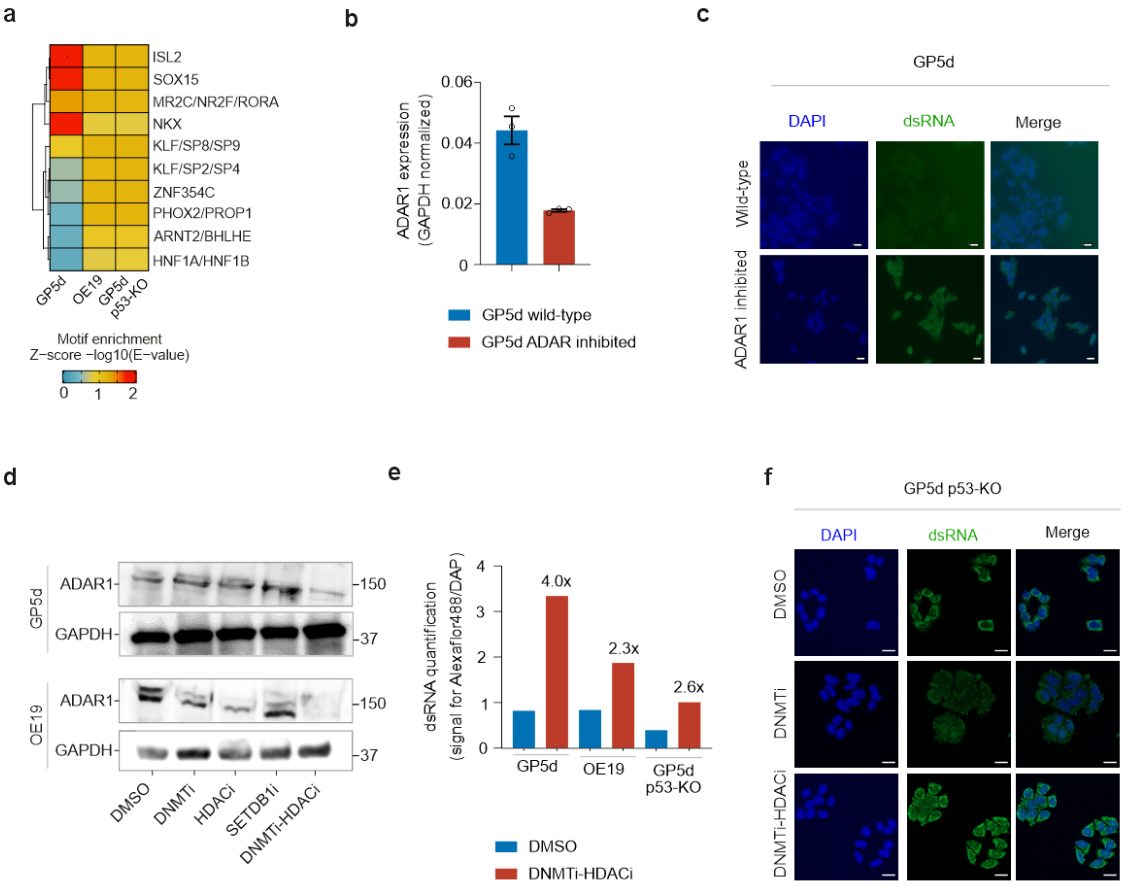

Supplementary Figure 10 | Inhibition of CMEs induces dsRNA expression. a, Motif enrichment analysis for IR-Alu SINEs in **Fig. 6a**. Motif enrichment analysis was performed as described in **Fig. 4a**. Hierarchical clustering was performed using Euclidean distance and Ward's method. **b,** qRT-PCR data showing the reduction in mRNA expression for ADAR1 in ADAR1 depleted GP5d cells. Y-axis shows GAPDH normalized gene expression. GP5d cells were transfected with Cas9 and ADAR1 sgRNA expressing plasmid and cells were selected for puromycin for 24 hours. The graph shows mean ± SD values for three replicates. **c,** Microscopy images for GP5d and ADAR1 depleted GP5d cells. DNA was stained with DAPI (blue), and dsRNA was stained using the J2 antibody (green). Scale bars are 20 μm. Cytoplasmic levels of dsRNA increased in ADAR1 inhibited GP5d cells. **d,** Western blot comparing ADAR1 expression for GP5d and OE19 cells treated with different CME inhibitors. For the ADAR1 blot in OE19 cells, the same cell lysate as in **Supplementary Fig. 2a** was used and thus the same GAPDH loading control was applied. The corresponding uncropped blots are presented in **Supplementary Fig. 20a**. **e,** Quantification of dsRNA in DMSO and DNMTi-HDACi treated GP5d, OE19 and GP5d p53-KO cells. The y-axis shows mean signal for (Alexa Flour 488/DAPI). The increase in dsRNA levels relative to DMSO control was shown for each cell line. **f,** Microscopy images for GP5d p53-KO cells treated with DMSO control, DNMTi and DNMTi-HDACi. DNA was stained with DAPI (blue) and dsRNA was stained using the J2 antibody (green). Scale bars are 20 μm. Source data are provided as **Supplementary Data 11**.

Supplementary Figure 11

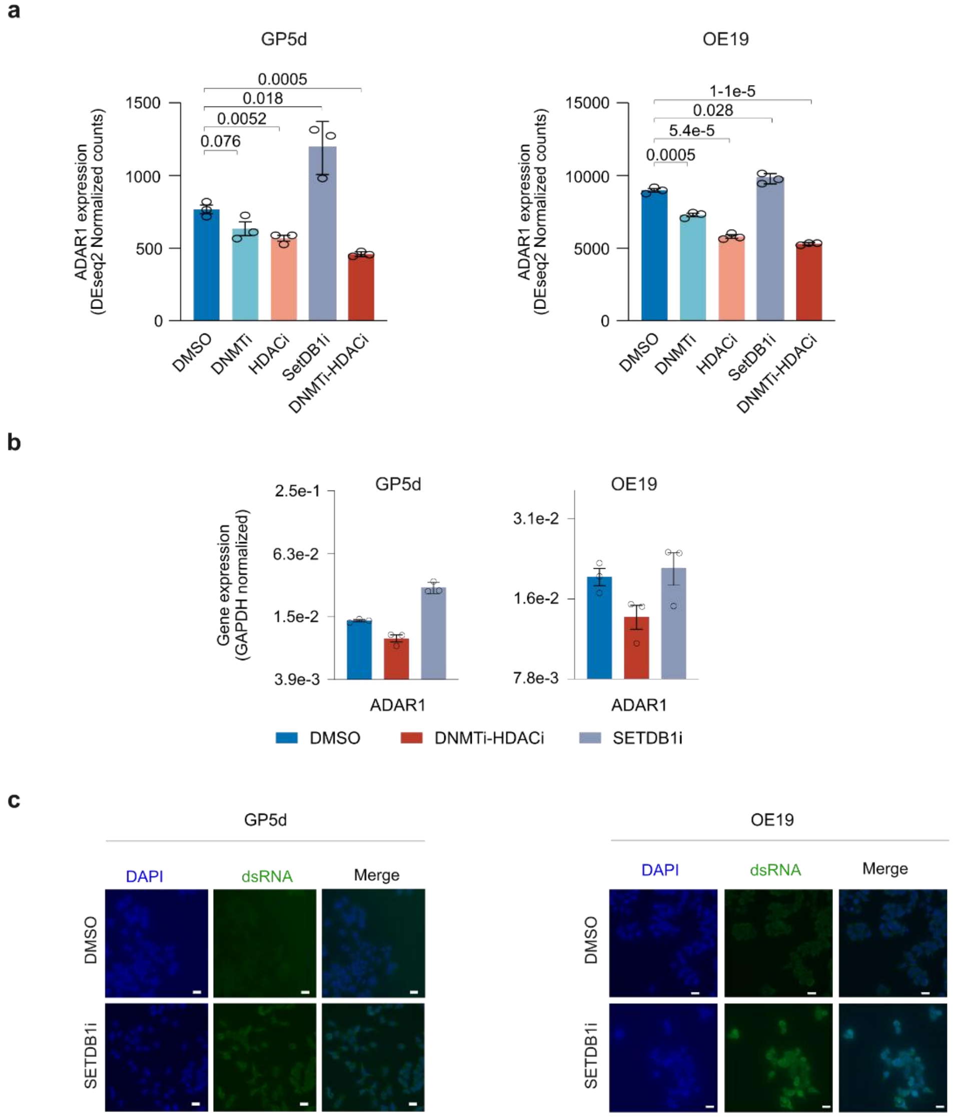

Supplementary Figure 11 | SETDB1 inhibition upregulates ADAR1 expression in GP5d and OE19 cells a, Comparison of normalized RNA-seq read counts for ADAR1 gene in GP5d, and OE19 cells treated with CME inhibitors. The graph shows mean ± SD values for three biological replicates (Unpaired two-sided t-test). **b,** qRT-PCR data showing ADAR1 mRNA expression in GP5d and OE19 cells treated with DMSO control, SETDB1i, and DNMTi-HDACi. Y-axis shows GAPDH normalized gene expression. The graph shows mean ± SD values for three replicates. **c,** Microscopy images for GP5d and OE19 cells treated with DMSO control or SETDB1i. DNA was stained with DAPI (blue), and dsRNA was stained using the J2 antibody (green). Scale bars are 20 µm. Cytoplasmic levels of dsRNA increased in SETDB1i treated GP5d and OE19 cells as compared to DMSO control. Source data are provided as **Supplementary Data 11**.

Supplementary Figure 12

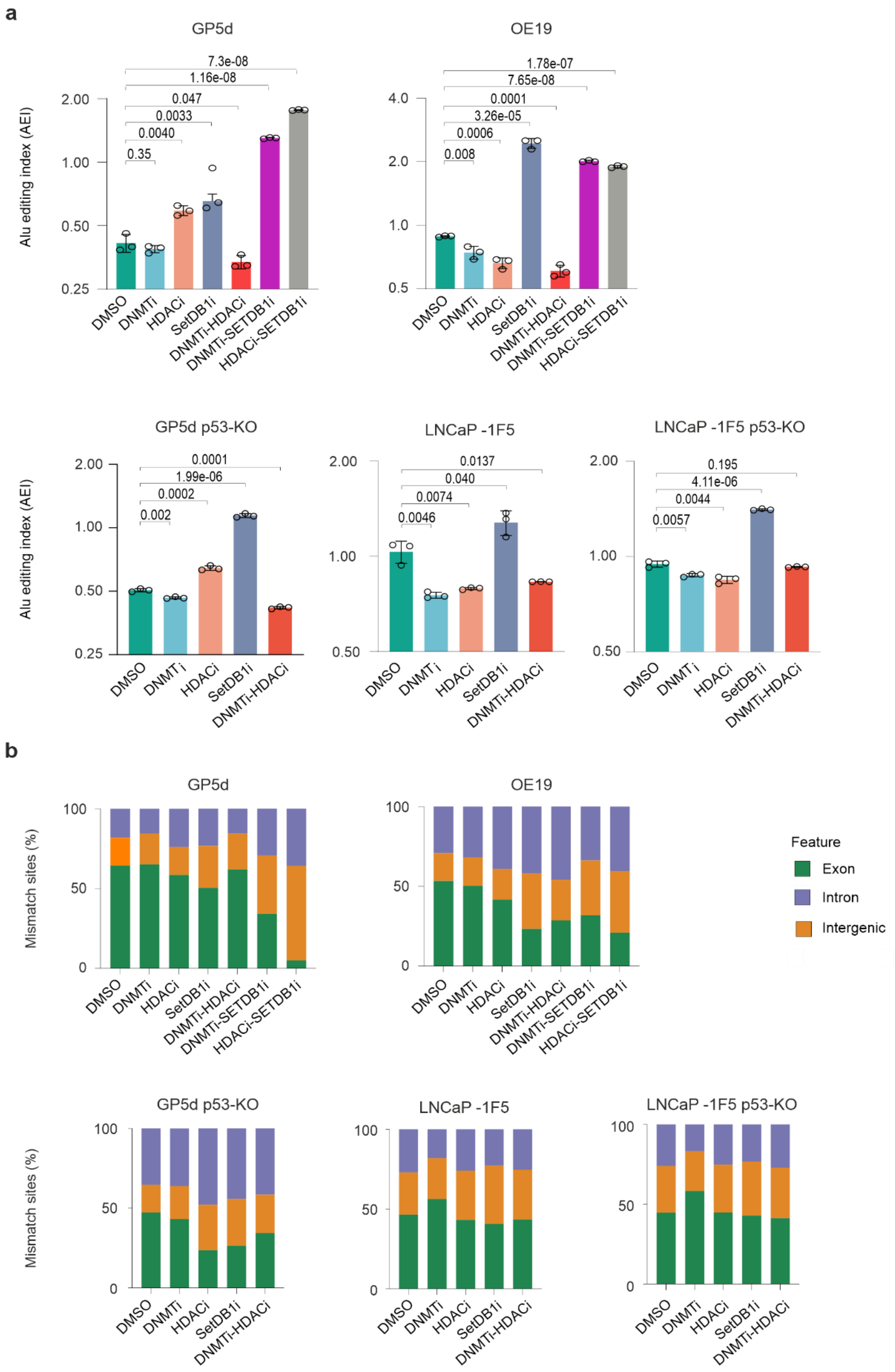

Supplementary Figure 12 | Distinct effects of CME treatments on Alu RNA editing. **a,** Bar plots comparing the Alu Editing Index (AEI) for GP5d, OE19, GP5d p53-KO, LNCaP-1F5, and LNCaP-1F5 p53-KO cells treated with different CMEs. The graph shows mean ± SD values for three biological replicates (unpaired two-sided t-test). **b,** Distribution of Alu editing sites in CME-treated GP5d, OE19, GP5d p53-KO, LNCaP-1F5, and LNCaP-1F5 p53-KO cells. Source data are provided as **Supplementary Data 11**.

Supplementary Figure 13

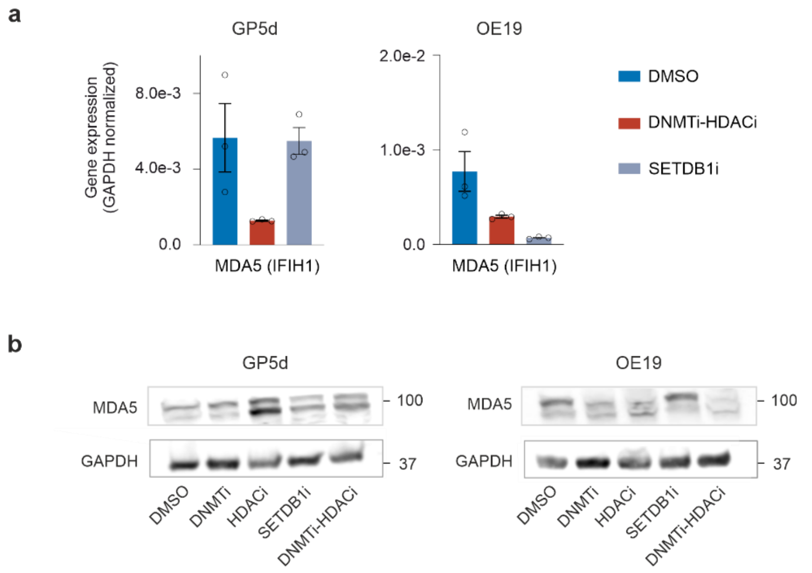

Supplementary Figure 13 | MDA5 expression in CMEi treated GP5d and OE19 cells. **a,** qRT-PCR data showing MDA5 (IFIH1) mRNA expression in GP5d and OE19 cells treated with DMSO control, SETDB1i and DNMTi-HDACi. Y-axis shows GAPDH normalized gene expression. The graph shows mean ± SD values for three replicates. **b,** Western blot of MDA5 expression for GP5d and OE19 cells treated with different CME inhibitors. Molecular weights in kDa are shown on the right side of the figures. For the MDA5 blot in GP5d and OE19 cells, the same cell lysate as in **Supplementary Fig. 2a** was used and thus the same GAPDH loading control was applied. The corresponding uncropped blots are presented in **Supplementary Fig. 20a**. Source data are provided as **Supplementary Data 11**.

Supplementary Figure 14

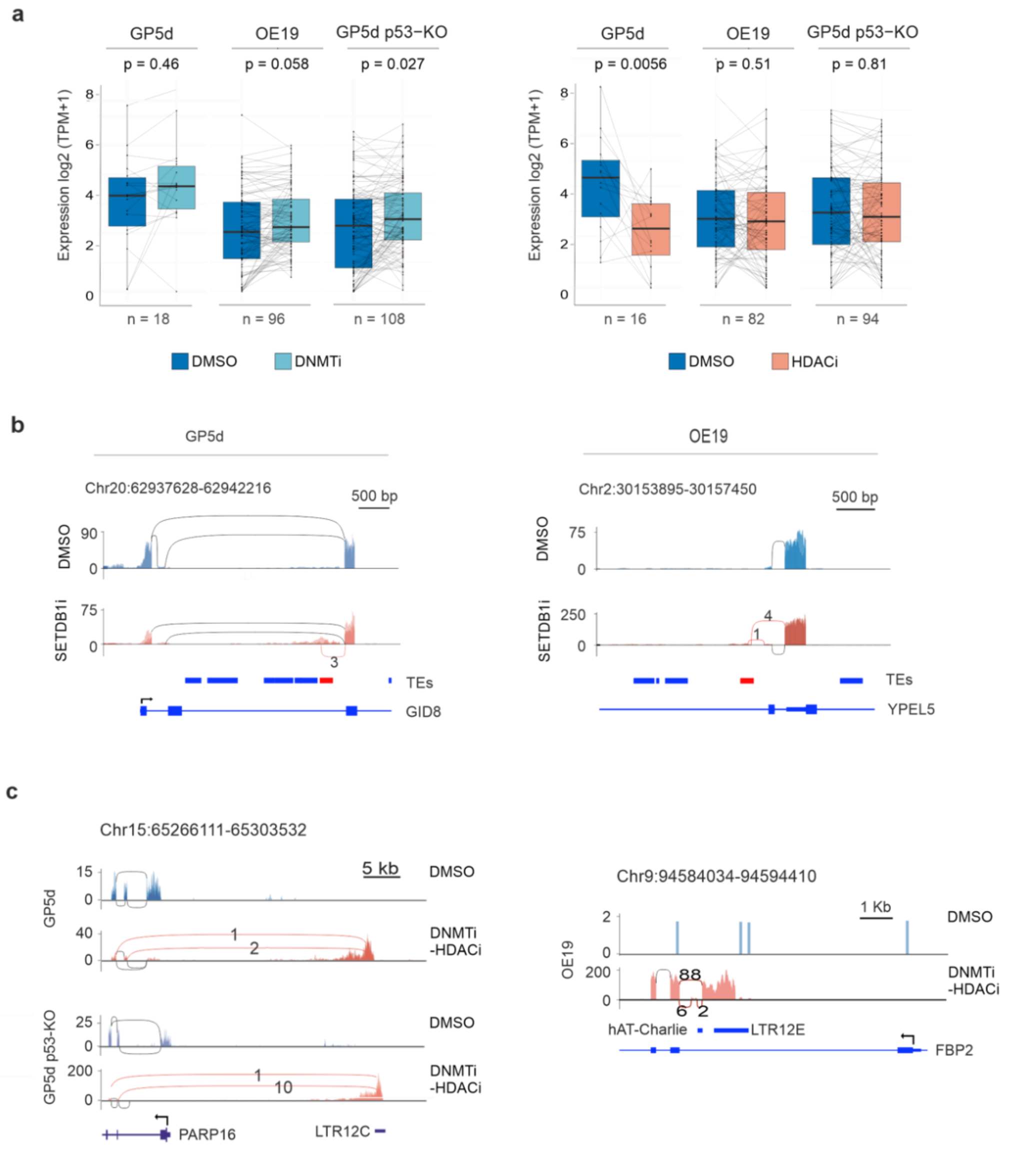

Supplementary Figure 14 | Comparison of CME inhibition-induced TE-chimeric transcript expression between three cell lines. a, DNMTi or HDACi increases the expression of TE-chimeric transcripts. Boxplots showing the expression of TE-chimeric transcripts in GP5d, OE19, and GP5d p53-KO cells with and without DNMTi and HDACi. P-values were calculated with a two-sided Wilcoxon test. Number of TE-chimeric transcripts expressed in at least two replicates in either DMSO control or inhibitor treatment shown for each group. **b,** Genome browser snapshot showing intronic Alu spliced into third exon of *GID8* gene to encode Alu-derived TE-chimeric transcript in SETDB1-inhibited GP5d. TE-chimeric transcripts from Alu SINE are highlighted in red with average number of TE-chimeric reads from three biological replicates indicated. Genome browser snapshot showing Alu spliced into exons of the *YPEL5* gene to encode Alu-derived TE-chimeric transcript from SETDB1-inhibited OE19 cells. **c,** Genome browser snapshot showing LTR12C spliced into the second and third exon of *PARP16* gene to encode LTR12C-derived TE-chimeric transcript in DNMTi-HDACi GP5d (upper left panel) and GP5d p53-KO cells (lower left panel panel). TE-chimeric transcripts from LTR12C are highlighted in red with the average number of TE-chimeric reads from three biological replicates indicated. Strong derepression of LTR12C in GP5d p53-KO cells contributes to higher number of TE-chimeric transcripts. Genome browser snapshot showing LTR12E spliced into second exon of the *FBP2* gene to encode LTR12E-derived TE-chimeric transcript from OE19 cells treated with DNMTi-HDACi. Source data are provided as **Supplementary Data 11**.

Supplementary Figure 15

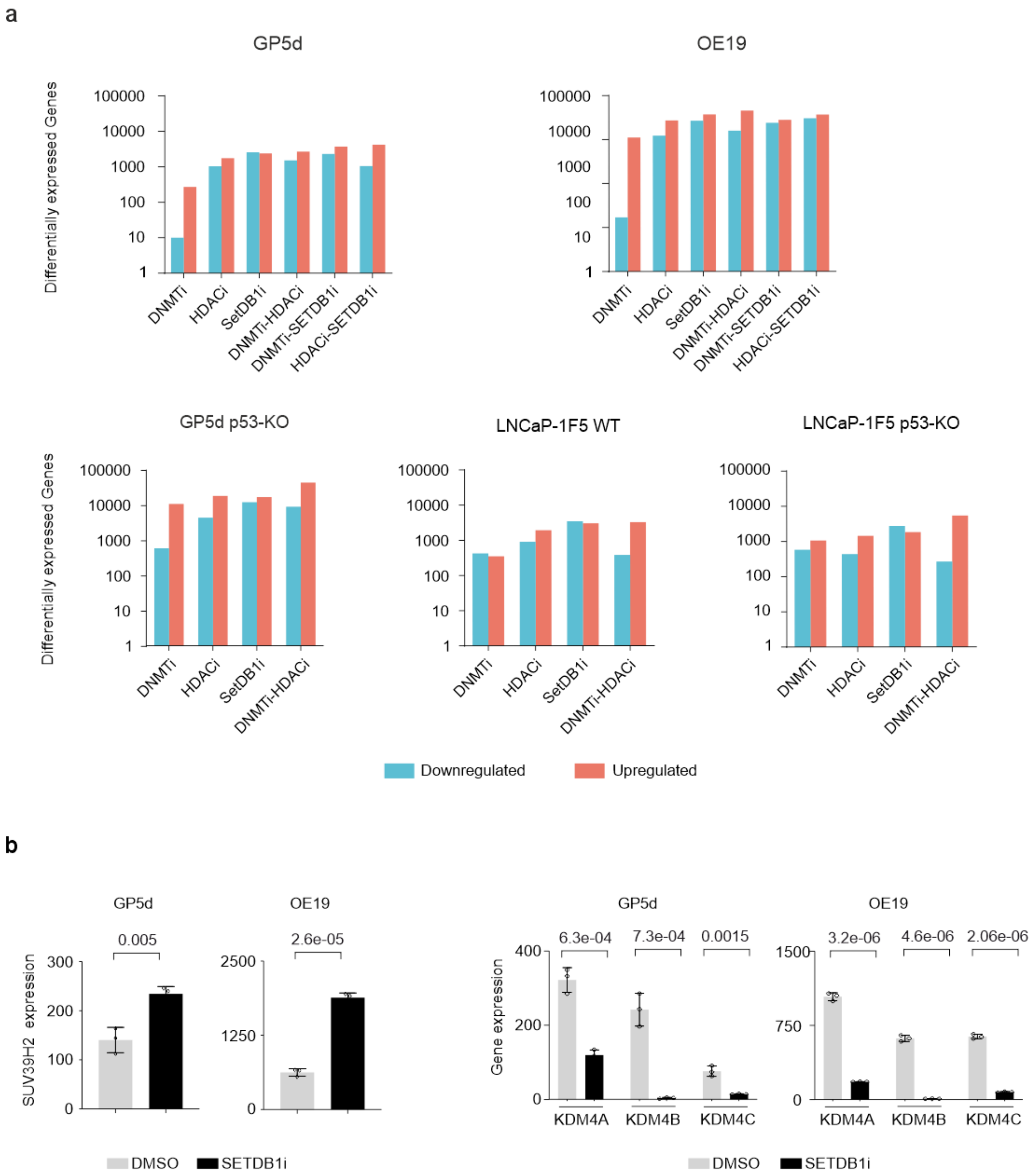

Supplementary Figure 15 | Loss of p53 is associated with stronger gene expression changes upon CME treatment. a, Number of differentially expressed genes (DEGs) by inhibition of CMEs in GP5d, OE19, GP5d p53-KO, LNCaP-1F5, and LNCaP-1F5 p53-KO cells. **b,** Bar plots comparing normalized RNA-seq read counts for SUV39H2 and KDM4A-C in GP5d and OE19 cells treated with SETDB1i or DMSO control. The graph shows mean ± SD values for three biological replicates (unpaired two-sided t-test). Source data are provided as **Supplementary Data 11**.

Supplementary Figure 16

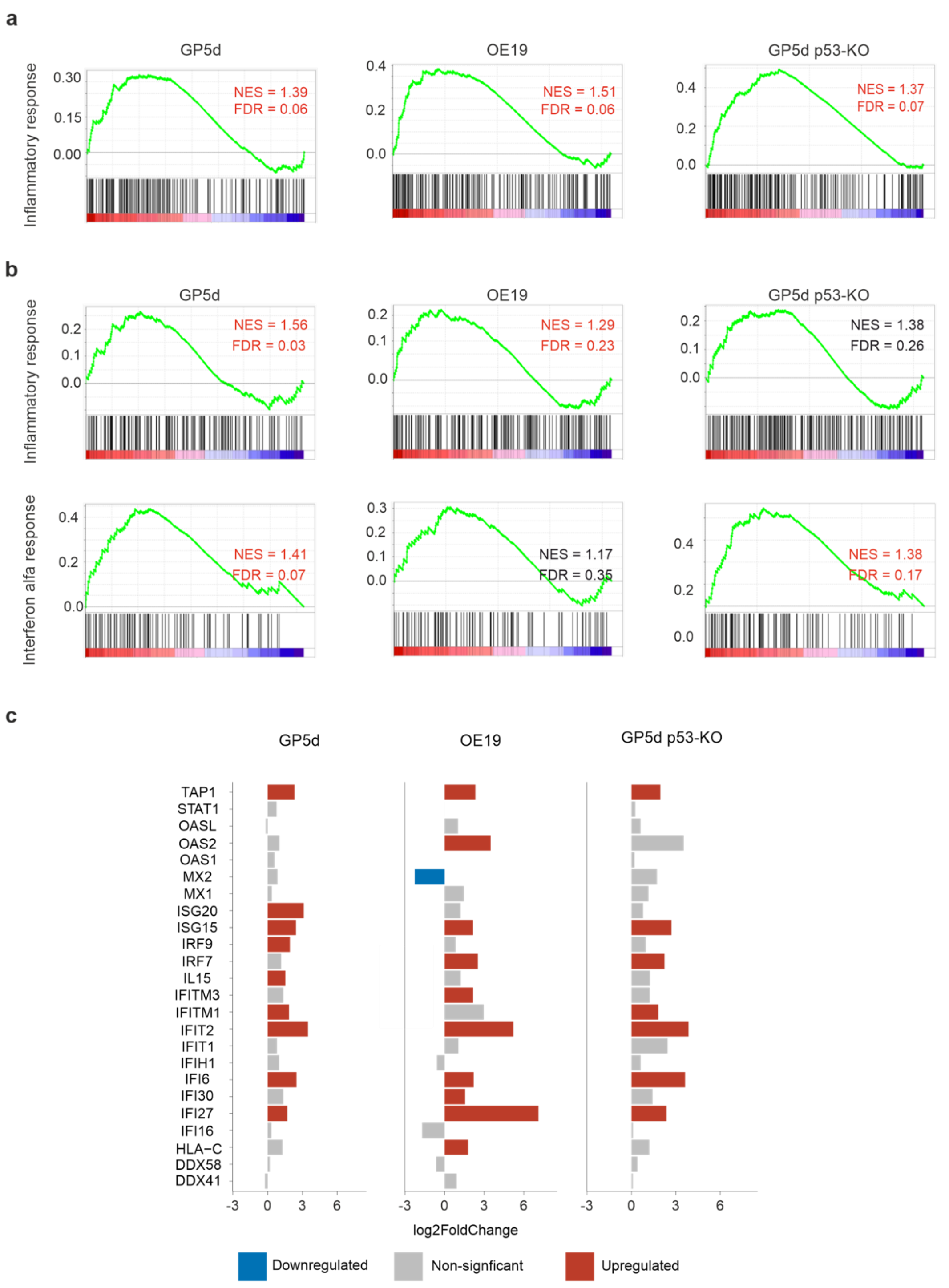

Supplementary Figure 16 | Inhibition of SETDB1 and co-inhibition of DNMT and HDAC activate inflammatory responses. a, GSEA for inflammatory response pathway enrichment in DNMTi-HDACi treated GP5d, OE19, and GP5d p53-KO cells. Normalized enrichment score (NES) and FDR for each pathway (FDR < 0.25 highlighted in red) are on the top-right corner. **b,** GSEA for enrichment of inflammatory response and interferon alpha response pathway in SETDB1 inhibited GP5d, OE19, and GP5d p53-KO cells. Normalized enrichment score (NES) and FDR for each pathway (FDR < 0.25 highlighted in red) are on top-right corner. **c,** Interferon stimulated genes are upregulated by SETDB1i treatment. Log2 fold changes in interferon stimulated genes were plotted for SETDB1i treated GP5d, OE19 and GP5d p53-KO cells. Source data are provided as **Supplementary Data 11**.

Supplementary Figure 17

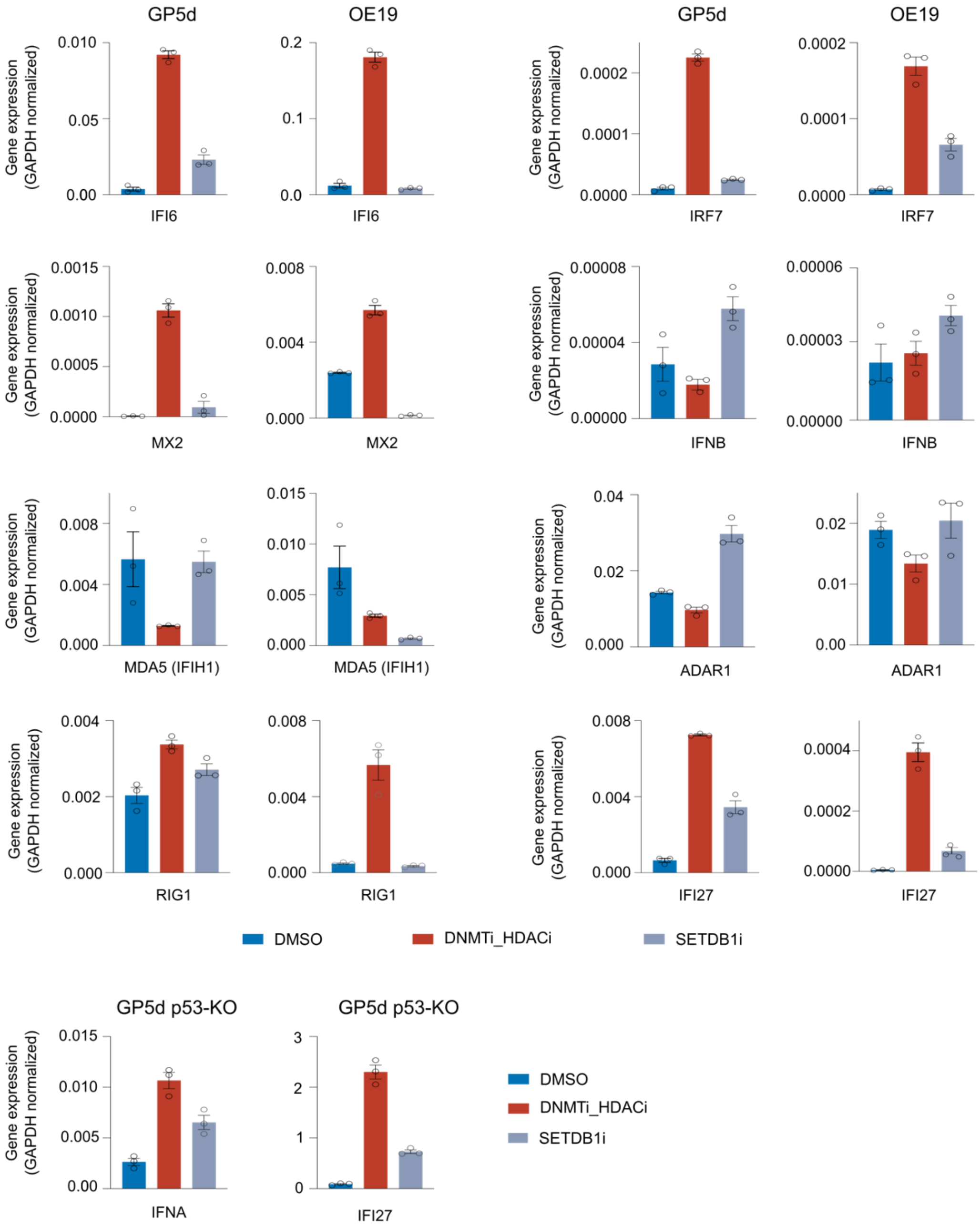

Supplementary Figure 17 | Inhibition of SETDB1 and co-inhibition of DNMT and HDAC induces the expression of interferon stimulated genes. qRT-PCR data showing the mRNA expression of interferon stimulated genes in GP5d, OE19 and GP5d p53-KO cells treated with DMSO control, SETDB1i and DNMTi-HDACi. Y-axis shows GAPDH normalized gene expression. The graph shows mean ± SD values for three replicates. Source data are provided as **Supplementary Data 11**.

Supplementary Figure 18

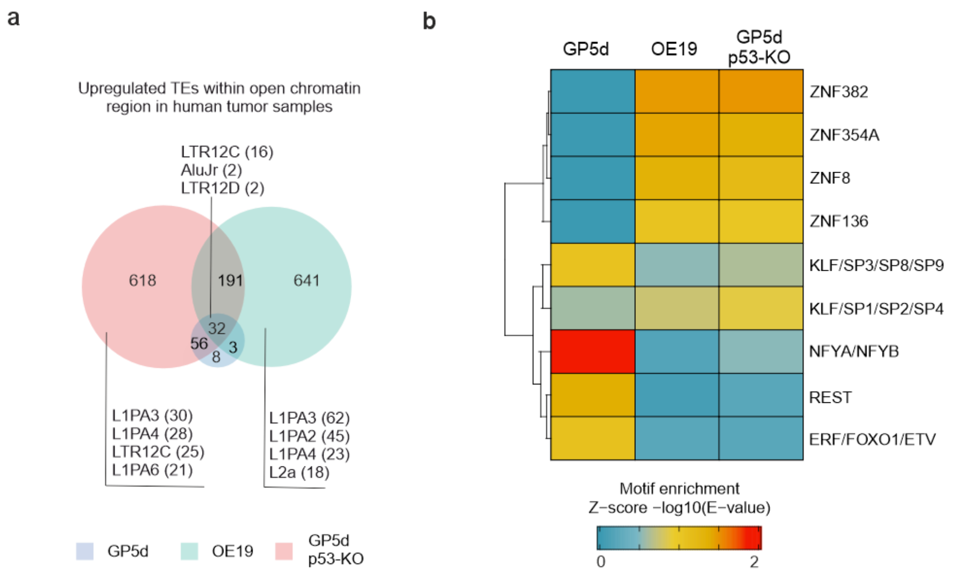

Supplementary Figure 18 | Cell type-specificity of the TEs derepressed by co-inhibition of DNMT and HDAC in colon and esophageal cancer cells shows open chromatin profile in tumor samples. a, Overlap analysis of TEs derepressed by DNMTi-HDACi treatment in GP5d, OE19, and GP5d p53-KO cells within open chromatin regions in human tumor samples of the respective cancer types. TCGA ATAC-seq peaks for colon adenocarcinoma (COAD) and esophageal carcinoma (ESCA) were extracted from ref. [8]. Venn diagram showing overlap between derepressed TEs with open chromatin profile in tumor samples. **b,** Motif enrichment analysis for TEs derepressed by DNMTi-HDACi treatment in GP5d, OE19, and GP5d p53-KO cells and having an open chromatin profile in tumor samples shown in Supplementary Fig. 18a. Motif enrichment analysis was performed as described in **Fig. 4a**. Clustering was performed using Euclidean distance and Ward's method. Source data are provided as **Supplementary Data 11**.

Supplementary Figure 19

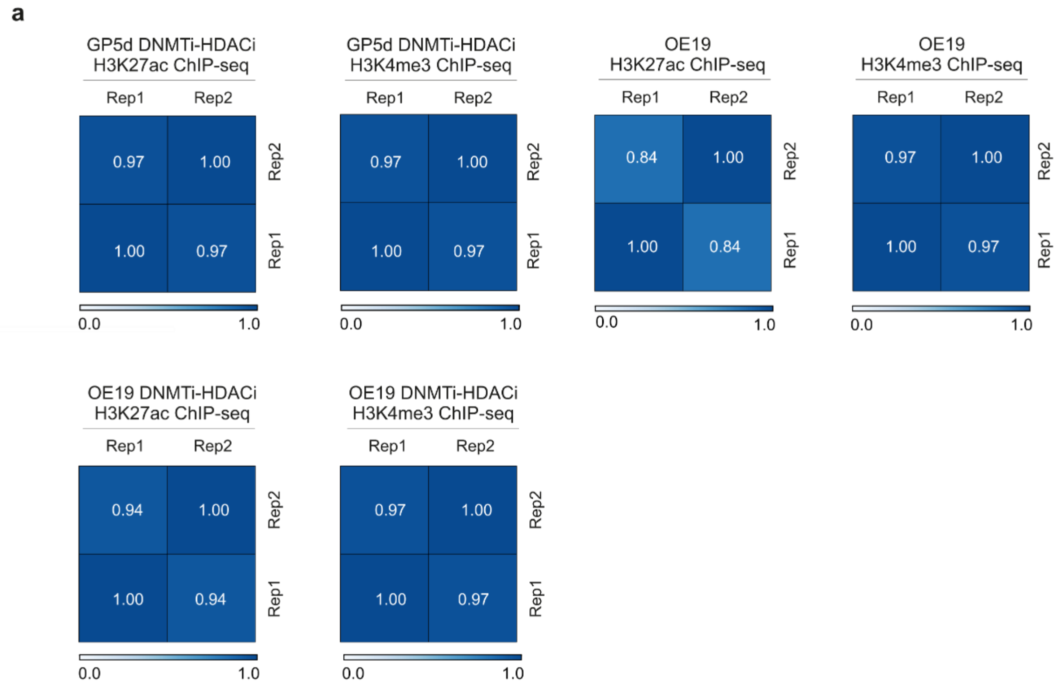

Supplementary Figure 19 | Quality control of ChIP-seq samples. **a,** Correlation plots for replicates of ChIP-seq for H3K27ac, H3K4me3 for GP5d and OE19 cells with and without DNMTi-HDACi. Pearson's r is shown in the heatmap figures.

Supplementary Figure 20

a

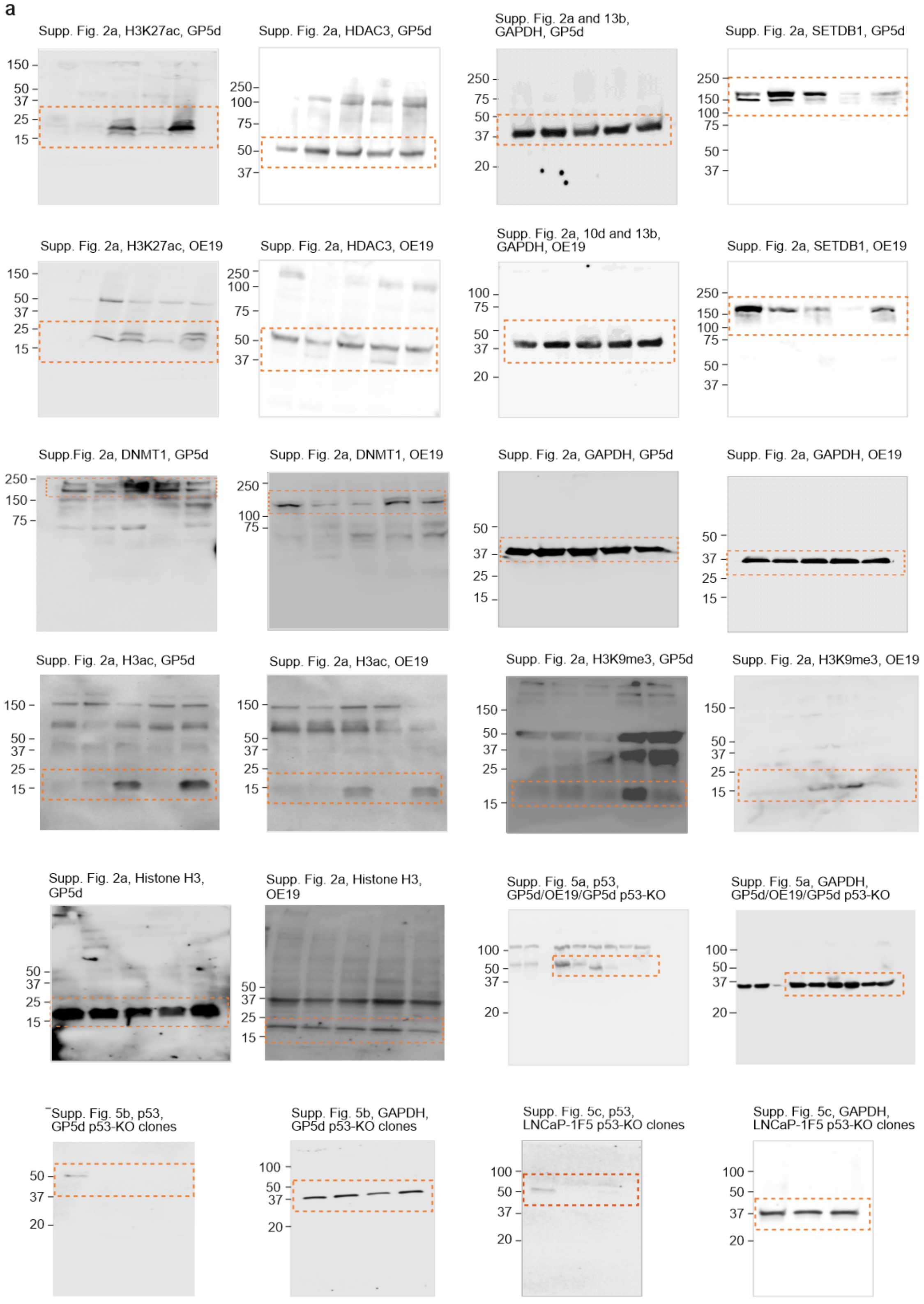

Supplementary Figure 20, continues

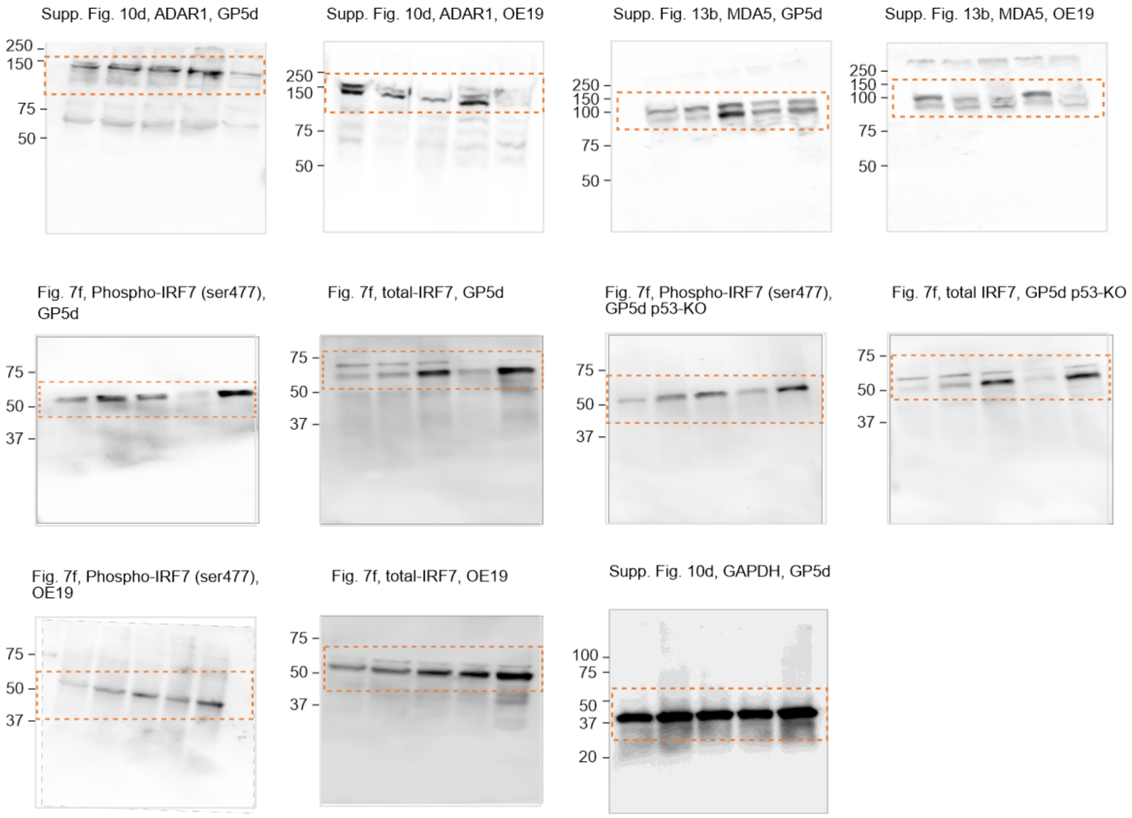

Supplementary Figure 20 | Source data for the western blots. a, Uncropped western blot images presented in the main and supplementary figures.

Supplementary Figure 21

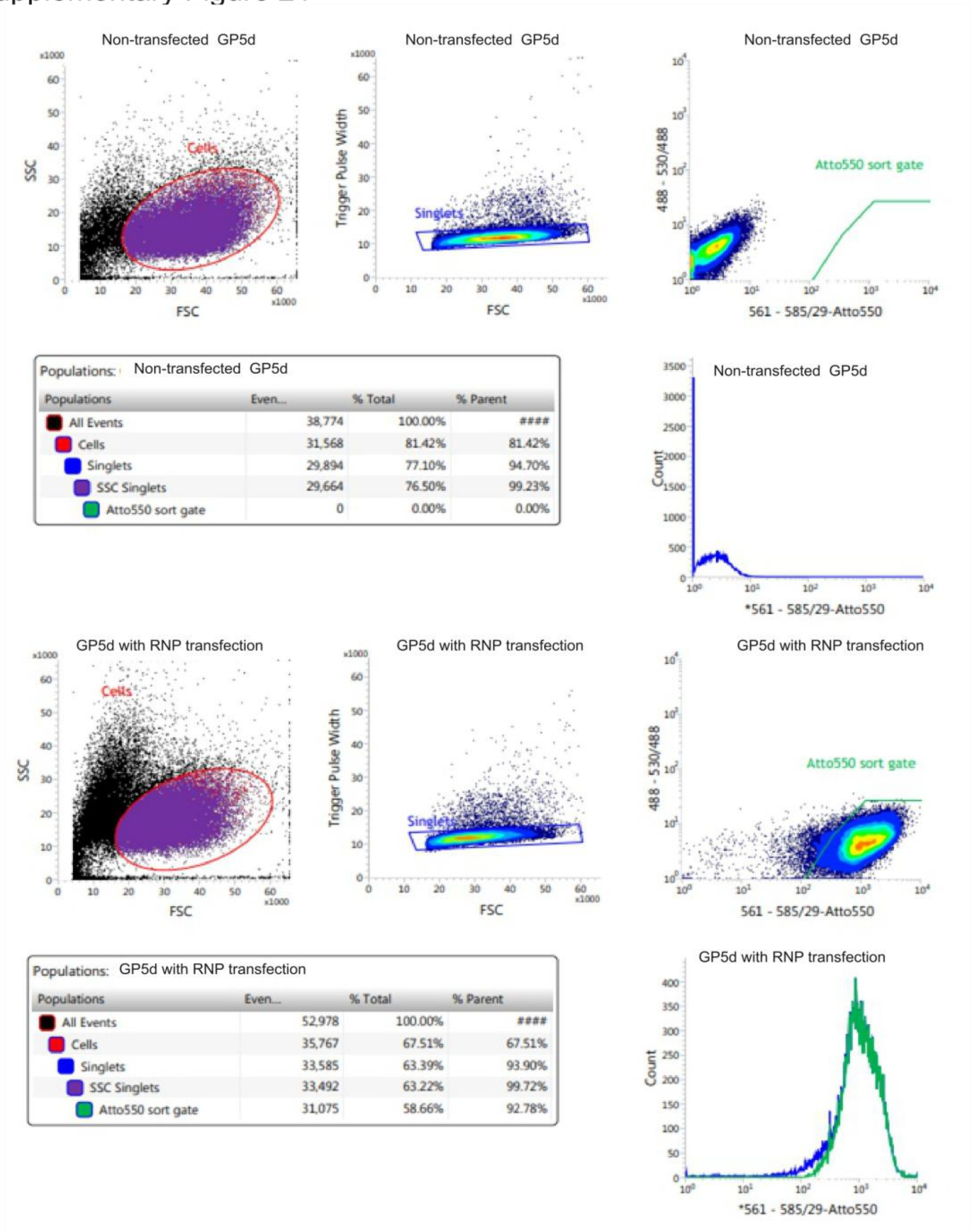

Populations: Non-transfected GP5d

Populations	Even...	% Total	% Parent
All Events	38,774	100.00%	####
Cells	31,568	81.42%	81.42%
Singlets	29,894	77.10%	94.70%
SSC Singlets	29,664	76.50%	99.23%
Atto550 sort gate	0	0.00%	0.00%

Populations: GP5d with RNP transfection

Populations	Even...	% Total	% Parent
All Events	52,978	100.00%	####
Cells	35,767	67.51%	67.51%
Singlets	33,585	63.39%	93.90%
SSC Singlets	33,492	63.22%	99.72%
Atto550 sort gate	31,075	58.66%	92.78%

Supplementary Figure 21 | Flow cytometry analysis for RNP transfected GP5d and LNCaP-1F5 cells. Flow cytometry analysis was used for measuring the RNP transfection efficiency of GP5d and LNCaP-1F5 cells and for sorting single ATTO550-positive cells on 96-well plates for generating clonal cell lines for p53 deletion, as tracrRNA used for generating the RNP complexes contain the ATTO550 fluorochrome. Representative flow cytometry analysis results for GP5d cells: Manual gating was performed using non-transfected GP5d cells (top panel), and similar gates were applied for RNP-transfected samples (lower panel represents GP5d transfected with RNP targeting p53). Gating strategy from left to right: 1. FSC/SSC: Cells were gated on the main population to exclude clear outliers such as cell debris. 2. FSC/Trigger pulse width: Cells were gated on the main population that represent single cells, excluding the outliers with larger trigger pulse width representing potential duplets. 3. Fluorescence was monitored on two channels: excitation 488 nm, emission 530/40 nm as an extra negative control, and excitation 561 nm, emission 585/29 nm for ATTO550. Gate was set using the non-transfected GP5d cells so that all cells remained negative for ATTO550. The same gate was maintained for analysis and sorting of RNP transfected cells.

Supplementary References:

1. Pawlak, A., Chybicka, K., Ziolo, E., Strzadala, L. & Kalas, W. The Contrasting Delayed Effects of Transient Exposure of Colorectal Cancer Cells to Decitabine or Azacitidine. *Cancers (Basel)* **14**(2022).
2. Chen, J. *et al.* Pracinostat (SB939), a histone deacetylase inhibitor, suppresses breast cancer metastasis and growth by inactivating the IL-6/STAT3 signalling pathways. *Life Sci* **248**, 117469 (2020).
3. Federico, A. *et al.* Mithramycin A and Mithralog EC-8042 Inhibit SETDB1 Expression and Its Oncogenic Activity in Malignant Melanoma. *Mol Ther Oncolytics* **18**, 83-99 (2020).
4. Fichter, C.D. *et al.* Occurrence of multipolar mitoses and association with Aurora-A/-B kinases and p53 mutations in aneuploid esophageal carcinoma cells. *BMC Cell Biol* **12**, 13 (2011).
5. Wang, T. *et al.* Species-specific endogenous retroviruses shape the transcriptional network of the human tumor suppressor protein p53. *Proc Natl Acad Sci U S A* **104**, 18613-8 (2007).
6. Jolma, A. *et al.* DNA-binding specificities of human transcription factors. *Cell* **152**, 327-39 (2013).
7. Vierstra, J. *et al.* Global reference mapping of human transcription factor footprints. *Nature* **583**, 729-736 (2020).
8. Corces, M.R. *et al.* The chromatin accessibility landscape of primary human cancers. *Science* **362**(2018).
