# Supplementary material for: Cancer cell type-specific derepression of transposable elements by inhibition of chromatin modifier enzymes
Source: Commun Biol. 2025 Jul 3;8:992. doi: 10.1038/s42003-025-08413-0 (PMC12229592; doi:10.1038/s42003-025-08413-0)
Supplement: Supplementary file 3 — Description of Additional Supplementary Files [file 42003_2025_8413_MOESM3_ESM.pdf]

Description of Supplementary Files:

1. **Patel_et_al_Supplementary_Figures.pdf :** Supplementary Figures and their legends as a single PDF.

2. **Patel_et_al_Supplementary_Data_1-10.xlsx :** Supplementary Data Tables 1-10 as individual sheets of an Excel file.

   Supplementary Data 1: Number of differentially expressed TE subfamilies by inhibition of CMEs as shown in **Supplementary Fig. 2b**.

   Supplementary Data 2: Fold-change (Log2) for TE subfamilies differentially expressed in at least one CME treatment in at least one cell line shown in **Fig. 1c** (treatment vs vehicle-treated cells from the same cell, absolute Log2FC > 2.5 and adjusted p-value < 0.05).

   Supplementary Data 3: Percentage of derepressed TE loci by inhibition of CMEs or SETDB1 KO shown in **Fig. 2b**, and Supplementary Fig. 6c.

   Supplementary Data 4: Number of LINEs and LTRs with p53RE elements. Expressed TEs with p53REs were stratified into five grades from least to most likely p53REs with transactivation potential using p53retriever shown in **Fig. 3g**.

   Supplementary Data 5: Fold-change (Log2) for TE subfamilies differentially expressed by SETDB1i/SETDB1-KO in at least one cell lines shown in **Fig. 6a** (treatment vs vehicle-treated cells from the same cell, absolute Log2FC >1.5 and adjusted p-value < 0.05).

   Supplementary Data 6: Expression counts (TPM) for TE-chimeric transcripts in CMEi treated/SETDB1i KO Cells. TE-chimeric transcripts expressed in at least two replicates in either control or treatment samples were used for analysis. TE-chimeric expression counts were used in **Fig. 7a-d**, and **Supplementary Fig. 14a**.

   Supplementary Data 7: Mutation status of chromatin modifier enzymes and ADAR gene in GP5d, OE19 and LNCaP cells.

   Supplementary Data 8: All used data and annotations in the study, including publications and accessions for GEO/ENCODE.

   Supplementary Data 9: List of guide RNAs and primers.

   Supplementary Data 10: Comparison of CME inhibitor treatments in distinct cell lines.

3. **Patel_et_al_Supplementary_Data_11.xlsx :** an Excel file containing source data for all graphs.
