# Supplementary material for: Cancer cell type-specific derepression of transposable elements by inhibition of chromatin modifier enzymes
Source: Commun Biol. 2025 Jul 3;8:992. doi: 10.1038/s42003-025-08413-0 (PMC12229592; doi:10.1038/s42003-025-08413-0)
Supplement: Supplementary file 6 — Reporting summary [file 42003_2025_8413_MOESM6_ESM.pdf]

nature portfolio

Corresponding author(s): Biswajyoti Sahu

Last updated by author(s): Jun 10, 2025

Reporting Summary

Nature Portfolio wishes to improve the reproducibility of the work that we publish. This form provides structure for consistency and transparency in reporting. For further information on Nature Portfolio policies, see our Editorial Policies and the Editorial Policy Checklist.

Statistics

For all statistical analyses, confirm that the following items are present in the figure legend, table legend, main text, or Methods section.

n/a	Confirmed	
☐	☒	The exact sample size (*n*) for each experimental group/condition, given as a discrete number and unit of measurement
☐	☒	A statement on whether measurements were taken from distinct samples or whether the same sample was measured repeatedly
☐	☒	The statistical test(s) used AND whether they are one- or two-sided<br>*Only common tests should be described solely by name; describe more complex techniques in the Methods section.*
☒	☐	A description of all covariates tested
☐	☒	A description of any assumptions or corrections, such as tests of normality and adjustment for multiple comparisons
☐	☒	A full description of the statistical parameters including central tendency (e.g. means) or other basic estimates (e.g. regression coefficient) AND variation (e.g. standard deviation) or associated estimates of uncertainty (e.g. confidence intervals)
☐	☒	For null hypothesis testing, the test statistic (e.g. *F*, *t*, *r*) with confidence intervals, effect sizes, degrees of freedom and *P* value noted<br>*Give P values as exact values whenever suitable.*
☒	☐	For Bayesian analysis, information on the choice of priors and Markov chain Monte Carlo settings
☒	☐	For hierarchical and complex designs, identification of the appropriate level for tests and full reporting of outcomes
☒	☐	Estimates of effect sizes (e.g. Cohen's *d*, Pearson's *r*), indicating how they were calculated

Our web collection on statistics for biologists contains articles on many of the points above.

Software and code

Policy information about availability of computer code

Data collection	No specialized software was used for data acquisition.
Data analysis	FastQC v.0.11.9 was used for quality control and determination of read lengths.<br><br>In the ChIP-seq and ATAC-seq data, Bowtie2 v.2.4.1 was used for mapping, Picard v.2.23.4 was used for marking duplicates and insert size analysis, Samtools v.1.7 was used for filtering reads, MACS2 v2.2.7.1 was used for peak calling, bedtools v.2.29.2 was used to subtract ENCODE blacklisted regions and calculating coverage and deepTools v.3.5.0 was used to create a RPKM-normalized coverage file. For ATAC-seq, reads mapped to the mitochondrial genome were removed with removeChrom.py script (https://github.com/jsh58/harvard/blob/master/removeChrom.py). Correlation analysis between ChIP-seq replicates was performed by using multiBigwigSummary v.3.1.3 and plotted with plotCorrelation v.3.1.3.<br><br>For KAS-seq and CUT&TAG data, Bowtie2 v.2.4.1 was used for mapping, Picard v.2.23.4 was used for marking duplicates, Samtools v.1.7 was used for filtering reads. DeepTools v.3.5.0 was used to create a RPKM-normalized coverage file.<br><br>RNA-seq reads were aligned to the human reference genome using STAR aligner v.2.7.5c with default parameters. Gene counts were quantified by using HTSeq count v.0.11.2. DESeq2 v.1.32.0 was used to identify differential expressed genes for each CMEs treatment as compared to DMSO control.<br><br>TEtranscripts v.2.2.1 was run on the SQuIRE alignment output. Output from DESeq2 v1.32.0 were used for TE subfamily expression analysis. Telescope v.1.0.3.1 analysis was performed on SQuIRE alignment output. Output from DESeq2 v1.32.0 were used in subsequent analysis. Nearby genes were associated with GREAT v.4.0.4.

Bwtools v.1.0 was used to plot compiled signal for ChIP-seq and KAS-seq signal at LTR12C consensus sequences.

All motif enrichments for derepressed TE loci were analyzed with AME from the MEME suite v. 5.0.2.

deepTools v.3.5.0 computeMatrix was used to compute a read matrix for the RPKM normalized bigwig files.

Statistical analysis was performed in R v.4.1.2. Genomic annotation for TE loci was performed with ChIPseeker v.1.28.3. All plotting was performed with ggplot2 v.3.3.6 from the Tidyverse suite v.1.3.1 94. GraphPad Prism v.9 (GraphPad) was used for Statistical analysis. Microscopy images were analysed with ZEISS ZEN v.3.10.

No custom software were used.

For manuscripts utilizing custom algorithms or software that are central to the research but not yet described in published literature, software must be made available to editors and reviewers. We strongly encourage code deposition in a community repository (e.g. GitHub). See the Nature Portfolio guidelines for submitting code & software for further information.

Data

Policy information about availability of data

All manuscripts must include a data availability statement. This statement should provide the following information, where applicable:

- Accession codes, unique identifiers, or web links for publicly available datasets
- A description of any restrictions on data availability
- For clinical datasets or third party data, please ensure that the statement adheres to our policy

Data generated in this study has been deposited in the GEO database under accession GSE254242.

GP5d ChIP-seq data for H3K27ac (GSM5454417), H3K27me3 (GSM5454428), and p53 (GSM5454412) were acquired from GEO database under accession GSE180158. GP5d ATAC-seq (GSE221051), NaNOMe-seq (“GSM7024433”), ChIP-seq for H3K4me3 (GSM6841187), rabbit IgG (GSM6841190) and mouse IgG (GSM6841189), and RNA-seq for DMSO treated (GSM6841203, GSM6841204, GSM6841205) and DNMTi-HDACi treated GP5d cells (GSM6841206, GSM6841207, GSM6841208) was acquired from GEO database under accession GSE221053. GP5d H3K4me1 (GSM1240814) was obtained with the GEO accession GSE51234. OE19 ATAC-seq (ERR1698333) was downloaded with accession code ERX1767841.

OE19 KAS-seq (ERR8135308) was acquired from accession code E-MTAB-11356. OE19 CUT&TAG data for H3K27me3 (ERR8105268), H3K4me1 (ERR8105270), RNA PolII (ERR8105275) and RNA PolIISp5 (ERR8105277) was obtained from ENA accession E-MTAB-11356.

A375 RNA-seq (GSM5320279, GSM5320280, GSM5320281) and A375 SETDB1-KO RNA-seq (GSM5320275, GSM5320276, GSM5320277) was acquired from GEO accession GSE155972.

A gene annotation GTF file was acquired from Gencode Release 36 for the reference chromosomes (https://ftp.ebi.ac.uk/pub/databases/gencode/Gencode_human/release_36/gencode.v36.annotation.gtf.gz). The GTF file was transferred into a BED file with gtfToBed.sh (github.com/timplab/nanoNOMe/blob/isac/analysis/annotations/gtfToBed.sh) and a TSS and a gene body BED files were created with a script adapted from https://github.com/isaclee/nanoNOMe/blob/master/snakemake/downloaded_data_parse.smk.

A repeatMasker.txt (2021-09-03) file was downloaded from the UCSC table browser (https://genome.ucsc.edu/cgi-bin/hgTables). LTR12C consensus sequences were acquired from RepBase (https://www.girinst.org/repbase/update/index.html).

GRCh38 chromosome sizes file (2020-03-13) file was downloaded from UCSC (https://hgdownload-test.gi.ucsc.edu/goldenPath/hg38/bigZips/latest/). GRCh38 blacklist BED file (ENCFF356LFX, release 2020-05-05) was downloaded from ENCODE project (https://www.encodeproject.org/).

Transcription factor motifs were acquired from JASPAR 2022 CORE non-redundant vertebrate annotations (https://jaspar.genereg.net/download/data/2022/CORE). The position weight matrices in MEME format were used for downstream motif analyses. Motif clustering data was downloaded from (https://resources.altius.org/~jvierstra/projects/motif-clustering-v2.0beta/).

TCGA cancer-type specific ATAC-seq peaks for colon adenocarcinoma (COAD) and esophageal carcinoma (ESCA) were acquired from https://gdc.cancer.gov/about-data/publications/ATACseq-AWG.

Research involving human participants, their data, or biological material

Policy information about studies with human participants or human data. See also policy information about sex, gender (identity/presentation), and sexual orientation and race, ethnicity and racism.

Reporting on sex and gender	N/A
Reporting on race, ethnicity, or other socially relevant groupings	N/A
Population characteristics	N/A
Recruitment	N/A

Ethics oversight	N/A

Note that full information on the approval of the study protocol must also be provided in the manuscript.

Field-specific reporting

Please select the one below that is the best fit for your research. If you are not sure, read the appropriate sections before making your selection.

☒ Life sciences ☐ Behavioural & social sciences ☐ Ecological, evolutionary & environmental sciences

For a reference copy of the document with all sections, see nature.com/documents/nr-reporting-summary-flat.pdf

Life sciences study design

All studies must disclose on these points even when the disclosure is negative.

Sample size	As standard practice of the field, all experiments were concluded on independent biological repeats as mentioned in the figure legends.
Data exclusions	ENCODE blacklisted regions were removed from all the peak files used in the analysis. Low-quality reads were filtered out from all used sequencing data.
Replication	All ChIP-seq were performed in two replicates. RNA-seq data was performed in triplicates. Pearson correlation analysis for ChIP-seq replicates shown in Supplementary Figure 19a.
Randomization	As experiments were performed on uniform biological material such commercial human cell lines, randomization of experimental groups was not applicable.
Blinding	Analysis was performed using large sequencing datasets representing whole human genome, thus blinding of the investigators was not relevant to this study.

Reporting for specific materials, systems and methods

We require information from authors about some types of materials, experimental systems and methods used in many studies. Here, indicate whether each material, system or method listed is relevant to your study. If you are not sure if a list item applies to your research, read the appropriate section before selecting a response.

Materials & experimental systems

n/a	Involved in the study	
☐	☒	Antibodies
☐	☒	Eukaryotic cell lines
☒	☐	Palaeontology and archaeology
☒	☐	Animals and other organisms
☒	☐	Clinical data
☒	☐	Dual use research of concern
☒	☐	Plants

Methods

n/a	Involved in the study	
☐	☒	ChIP-seq
☐	☒	Flow cytometry
☒	☐	MRI-based neuroimaging

Antibodies

Antibodies used	H3K4me3 (Diagenode, C15410003) and H3K27ac (Diagenode, C15410196).
Validation	The anti-H3K4me3 polyclonal antibody is raised in rabbit against the region of histone H3 containing trimethylated lysine 4. It is recommended for detecting H3K4me3 in ChIP-experiments in human by the manufacturer with >100 citations available for this antibody (https://www.diagenode.com/en/documents/datasheet-h3k4me3-C15410003).<br><br>The anti-H3K27ac polyclonal antibody is raised in rabbit against the region of histone H3 containing aceylation at lysine 27. It is recommended for detecting H3K27ac in ChIP-experiments in human by the manufacturer, and there is validation data and >80 citations available for this antibody (https://www.diagenode.com/en/p/h3k27ac-polyclonal-antibody-premium-50-mg-18-ml).

Eukaryotic cell lines

Policy information about cell lines and Sex and Gender in Research

Cell line source(s)	Colon cancer cell line GP5d (Sigma, 95090715) , OE19 (Sigma, 96071721) and LNCaP-1F5 (was available in lab, From study Sahu et. al 2011)

Authentication

Cell lines were directly obtained from trusted vendors (Sigma) and low-passage cells were used in experiments. Cell lines were not authenticated.

Mycoplasma contamination

All cell lines tested negative for mycoplasma contamination upon purchase and were routinely checked as per standard good laboratory practice.

Commonly misidentified lines
(See ICLAC register)

Cell lines used in this study are not in the list of commonly misidentified cell lines.

Plants

Seed stocks

N/A

Novel plant genotypes

N/A

Authentication

N/A

ChIP-seq

Data deposition

☒ Confirm that both raw and final processed data have been deposited in a public database such as GEO.

☒ Confirm that you have deposited or provided access to graph files (e.g. BED files) for the called peaks.

Data access links
May remain private before publication.

Raw and processed files have been deposited into GEO under accession GSE254242.

Files in database submission

GP5d_DNMTi_HDACi_H3K27ac_Rep1_R1_001.fastq.gz
GP5d_DNMTi_HDACi_H3K27ac_Rep2_R1_001.fastq.gz
GP5d_DNMTi_HDACi_H3K4me3_Rep1_R1_001.fastq.gz
GP5d_DNMTi_HDACi_H3K4me3_Rep2_R1_001.fastq.gz
OE19_DNMTi_HDACi_H3K27ac_Rep1_R1_001.fastq.gz
OE19_DNMTi_HDACi_H3K27ac_Rep2_R1_001.fastq.gz
OE19_DNMTi_HDACi_H3K4me3_Rep1_R1_001.fastq.gz
OE19_DNMTi_HDACi_H3K4me3_Rep2_R1_001.fastq.gz
OE19_H3K27ac_Rep1_R1_001.fastq.gz
OE19_H3K27ac_Rep2_R1_001.fastq.gz
OE19_H3K4me3_Rep1_R1_001.fastq.gz
OE19_H3K4me3_Rep2_R1_001.fastq.gz
OE19_Input_R1_001.fastq.gz
GP5d_DNMTi_HDACi_H3K27ac_Rep1_peaks_blacklisted.narrowPeak
GP5d_DNMTi_HDACi_H3K27ac_Rep1_final_RPKM_normalized.bw
GP5d_DNMTi_HDACi_H3K27ac_Rep2_peaks_blacklisted.narrowPeak
GP5d_DNMTi_HDACi_H3K27ac_Rep2_final_RPKM_normalized.bw
GP5d_DNMTi_HDACi_H3K4me3_Rep1_peaks_blacklisted.narrowPeak
GP5d_DNMTi_HDACi_H3K4me3_Rep1_final_RPKM_normalized.bw
GP5d_DNMTi_HDACi_H3K4me3_Rep2_peaks_blacklisted.narrowPeak
GP5d_DNMTi_HDACi_H3K4me3_Rep2_final_normalized_RPKM.bw
OE19_DNMTi_HDACi_H3K27ac_Rep1_peaks_blacklisted.narrowPeak
OE19_DNMTi_HDACi_H3K27ac_Rep1_final_RPKM_normalized.bw
OE19_DNMTi_HDACi_H3K27ac_Rep2_peaks_blacklisted.narrowPeak
OE19_DNMTi_HDACi_H3K27ac_Rep2_final_RPKM_normalized.bw
OE19_DNMTi_HDACi_H3K4me3_Rep1_peaks_blacklisted.narrowPeak
OE19_DNMTi_HDACi_H3K4me3_Rep1_final_RPKM_normalized.bw
OE19_DNMTi_HDACi_H3K4me3_Rep2_peaks_blacklisted.narrowPeak
OE19_DNMTi_HDACi_H3K4me3_Rep2_final_RPKM_normalized.bw
OE19_H3K27AC_Rep_1_peaks_blacklisted.narrowPeak
OE19_H3K27ac_Rep1_final_RPKM_normalized.bw
OE19_H3K27Ac_Rep2_peaks_blacklisted.narrowPeak
OE19_H3K27ac_Rep2_final_RPKM_normalized.bw
OE19_H3K4me3_Rep1_peaks_blacklisted.narrowPeak
OE19_H3K4me3_Rep1_final_RPKM_normalized.bw
OE19_H3K4me3_Rep2_peaks_blacklisted.narrowPeak
OE19_H3K4me3_Rep2_final_RPKM_normalized.bw
OE19_Input_final_RPKM_normalized.bw

Genome browser session
(e.g. UCSC)

BigWig track files and peak files are deposited in GEO for loading into a genome browser.

Methodology

Replicates	Two biological replicates were used for ChIP-seq.
Sequencing depth	GP5d_DNMTi_HDACi_H3K27ac_Rep1_R1_001.fastq.gz, 20071220 reads<br>GP5d_DNMTi_HDACi_H3K27ac_Rep2_R1_001.fastq.gz, 22264604 reads<br>GP5d_DNMTi_HDACi_H3K4me3_Rep1_R1_001.fastq.gz, 37460207 reads<br>GP5d_DNMTi_HDACi_H3K4me3_Rep2_R1_001.fastq.gz, 20182612 reads<br>OE19_DNMTi_HDACi_H3K27ac_Rep1_R1_001.fastq.gz, 28159157 reads<br>OE19_DNMTi_HDACi_H3K27ac_Rep2_R1_001.fastq.gz, 28142269 reads<br>OE19_DNMTi_HDACi_H3K4me3_Rep1_R1_001.fastq.gz, 26018842 reads<br>OE19_DNMTi_HDACi_H3K4me3_Rep2_R1_001.fastq.gz, 26116919 reads<br>OE19_H3K27ac_Rep1_R1_001.fastq.gz, 13772158 reads<br>OE19_H3K27ac_Rep2_R1_001.fastq.gz, 25972931 reads<br>OE19_H3K4me3_Rep1_R1_001.fastq.gz, 29852516 reads<br>OE19_H3K4me3_Rep2_R1_001.fastq.gz, 27600112 reads<br>OE19_Input_R1_001.fastq.gz, 19254288 reads
Antibodies	H3K4me3 (Diagenode, C15410003) and H3K27ac (Diagenode, C15410196).
Peak calling parameters	Peaks were called using MACS2 with options -f BAM -g hs --keep-dup all
Data quality	Fastqc v.0.11.9 was used for quality control of raw data, alignment statistics were checked, fraction of reads in peaks (FRiP) was calculated and data was manually inspected in a genome browser.
Software	Bowtie2 v.2.4.1 (Langmead and Salzberg, Nat Methods 9, 357-359, 2012)<br>Samtools 1.7 (Li et al., Bioinformatics, 25(16): 2078-2079, 2009)<br>Picard Tools v.2.23.4 (http://broadinstitute.github.io/picard/)<br>MACS2 v.2.2.7.1 (Zhang et al. Genome Biol. 9, pp. R137, 2008)

Flow Cytometry

Plots

Confirm that:

☒ The axis labels state the marker and fluorochrome used (e.g. CD4-FITC).

☒ The axis scales are clearly visible. Include numbers along axes only for bottom left plot of group (a 'group' is an analysis of identical markers).

☒ All plots are contour plots with outliers or pseudocolor plots.

☒ A numerical value for number of cells or percentage (with statistics) is provided.

Methodology

Sample preparation	GP5d and LNCaP-1F5 cells were transfected with ribonucleoprotein (RNP) complex. Equimolar ratios of target-specific crRNAs and ATTO550-tracrRNA (IDT, 1075928) were annealed. RNP complex were constituted from Alt-R S.p. HiFi Cas9 Nuclease V3 (IDT, 1081060; 1,000ng per 200,000 cells) and target-specific sgRNA (250ng per 200,000 cells) and transfected to cells by using CRISPRMAX (Life Technologies, CMAX000003) according to manufacturer's protocol. Cells were trypsinized 24 hours after transfection, washed once and resuspended in cold PBS. The flow cytometry analysis at the HiLife Flow Cytometry Unit, University of Helsinki, Finland, using BD Influx System (USB) and BD FACS software (version 1.2.0.142).
Instrument	BD Influx System (USB), model number X646500S7001
Software	BD FACS™ Software, 1.2.0.142
Cell population abundance	Out of 35,767 RNP transfected GP5d cells analyzed, 93.90% were singlets based on SSC/FSC, out of which 99.72% were ssc-singlets excluding the outliers with larger trigger pulse width representing potential duplets. Gate for ATTO550 was set so that all non-transfected cells were negative. Out of 33,492 singlets cells analyzed from the transfected sample, 92.78% were positive for ATTO550.
Gating strategy	Manual gating was performed using non-transfected GP5d and LNCaP-1F5 cells, and similar gates were applied for RNP transfected samples to analyze transfection efficiency. Gating strategy is described in Extended Data Fig. 21 (top panels). Gating strategy from left to right: 1. FSC/SSC: Cells were gated on the main population to exclude clear outliers such as cell debris. 2. FSC/Trigger pulse width: Cells were gated on the main population that represent single cells, excluding the outliers with larger trigger pulse width representing potential duplets. 3. Fluorescence was monitored on two channels: excitation 488nm, emission 530/40nm as an extra negative control, and excitation 561nm, emission 585/29nm for ATTO550. Gate was

set using the non-transfected GP5d and LNCAP-1F5 cells so that all cells remained negative for ATTO550. Same gate was maintained to analyze RNP transfected cells to measure the proportion of ATTO550-positive cells.

☒ Tick this box to confirm that a figure exemplifying the gating strategy is provided in the Supplementary Information.
